# TMT-Based Plasma Proteomics Reveals Dyslipidemia Among Lowlanders During Prolonged Stay at High Altitudes

**DOI:** 10.3389/fphys.2021.730601

**Published:** 2021-10-15

**Authors:** Pooja, Vandana Sharma, Ram Niwas Meena, Koushik Ray, Usha Panjwani, Rajeev Varshney, Niroj Kumar Sethy

**Affiliations:** ^1^Peptide and Proteomics Division, Defence Institute of Physiology and Allied Sciences, New Delhi, India; ^2^Neurophysiology Department, Defence Institute of Physiology and Allied Sciences, New Delhi, India

**Keywords:** high altitude, hypobaric hypoxia, acclimatization, plasma proteomics, inflammation, dyslipidemia

## Abstract

Acute exposure to high altitude perturbs physiological parameters and induces an array of molecular changes in healthy lowlanders. However, activation of compensatory mechanisms and biological processes facilitates high altitude acclimatization. A large number of lowlanders stay at high altitude regions from weeks to months for work and professional commitments, and thus are vulnerable to altitude-associated disorders. Despite this, there is a scarcity of information for molecular changes associated with long-term stay at high altitudes. In the present study, we evaluated oxygen saturation (SpO_2_), heart rate (HR), and systolic and diastolic blood pressure (SBP and DBP) of lowlanders after short- (7 days, HA-D7) and long-term (3 months, HA-D150) stay at high altitudes, and used TMT-based proteomics studies to decipher plasma proteome alterations. We observed improvements in SpO_2_ levels after prolonged stay, while HR, SBP, and DBP remained elevated as compared with short-term stay. Plasma proteomics studies revealed higher levels of apolipoproteins APOB, APOCI, APOCIII, APOE, and APOL, and carbonic anhydrases (CA1 and CA2) during hypoxia exposure. Biological network analysis also identified profound alterations in lipoprotein-associated pathways like plasma lipoprotein assembly, VLDL clearance, chylomicron assembly, chylomicron remodeling, plasma lipoprotein clearance, and chylomicron clearance. In corroboration, lipid profiling revealed higher levels of total cholesterol (TC), triglycerides (TGs), low-density lipoprotein (LDL) for HA-D150 whereas high density lipoproteins (HDL) levels were lower as compared with HA-D7 and sea-level indicating dyslipidemia. We also observed higher levels of proinflammatory cytokines IL-6, TNFα, and CRP for HA-D150 along with oxidized LDL (oxLDL), suggesting vascular inflammation and proartherogenic propensity. These results demonstrate that long-term stay at high altitudes exacerbates dyslipidemia and associated disorders.

## Introduction

Human beings experience compromised oxygen delivery at high altitudes (≥ 2,500 m) due to decreased atmospheric pressure and partial pressure of oxygen. This condition of hypobaric hypoxia is the unavoidable, unmodifiable, and uniform environmental stress for everyone at any given altitude. In addition, extreme cold, solar radiation, and aridity are other major stressors for lowlanders at high altitudes, at least for the first few days upon the arrival. Lowlanders elicit an integrated physiological (including respiratory and cardio-pulmonary) and hematological response for acclimatization to high altitude (Houston and Riley, 1947; Bartsch and Gibbs, 2007; Naeije, 2010). It is generally accepted that increased ventilation and hemoconcentration facilitate near normalization of the arterial oxygen content of lowlanders after an approximately 7-day residence at a high altitude (Muza et al., 2010). More recently, several proteins and biochemical pathways have been reported facilitating high altitude acclimatization (Padhy et al., 2016; Tang et al., 2018; Gangwar et al., 2019; Pooja et al., 2020). Failure in such responses may lead to high altitude illnesses, ranging from acute mountain sickness (AMS) to severe and life-threatening forms like high altitude pulmonary edema (HAPE), high altitude cerebral edema (HACE), thromboembolism, and high altitude polycythemia (HAPC) (Reynafarje et al., 1959; Hackett and Roach, 2001; Gallagher and Hackett, 2004; Palmer, 2010; Gupta and Ashraf, 2012). Both animal and human studies have reported that hypobaric hypoxia-induced oxidative stress and inflammation are important factors for the genesis of these high altitude maladies (Hartmann et al., 2000; Sarada et al., 2008; Himadri et al., 2010; Siervo et al., 2014; Boos et al., 2016; Pena et al., 2020a; Pham et al., 2021).

More than 40 million people including thousands of army personnel, government officials, miners, pilgrims, trekkers, and porters visit high altitude regions annually and are at risk of suffering from altitude illness and potentially dying from it (Moore, 2001; Basnyat, 2018). The working conditions and duration of high altitude residence differ among lowlanders depending on their professional requirements. On average trekkers, porters, sportsperson, pilgrims, and tourists spend few days to weeks at high altitudes whereas military personnel, diplomats, miners, and scientists spend months to years at high altitudes. Both acute and chronic exposure to high altitude induces several metabolic alterations and adjustments (Murray, 2016; Murray et al., 2018). More specifically, hypobaric hypoxia exposure alters lipid profiles (Gangwar et al., 2019) inducing hypercholesterolemia and hypertriglyceridemia (Mohanna et al., 2006; Sherpa et al., 2011; Vats et al., 2013) and associated cardiovascular disorders above 3,000 m (Virues-Ortega et al., 2009; Mallet et al., 2021). In addition, extended stay of young and healthy lowlanders at high and extreme altitudes is reportedly associated with a higher risk of spontaneous vascular thrombosis (Anand et al., 2001), massive infarcts, and stroke (Jha et al., 2002). Hence, studying lowlander molecular response above 3,000 m and extended residence period (weeks to months) is a pertinent scientific endeavor that can save both money and manpower.

Blood plasma is a highly accessible sample for monitoring the health status of an individual (Geyer et al., 2016), and high-throughput plasma proteome analysis is a popular omics method to investigate system-level protein alterations that may be rooted from basic science or clinical perspective (Pernemalm and Lehtio, 2014; Ignjatovic et al., 2019). Several research groups including ours have used plasma proteomics for gaining insight into high altitude acclimatization (Ahmad et al., 2011, 2013; Levett et al., 2011, 2015; Julian et al., 2014; Yang et al., 2014; Lu et al., 2018; Wang et al., 2019) and high altitude disorders like AMS (Julian et al., 2014; Lu et al., 2018), HAPE (Ahmad et al., 2011; Yang et al., 2014), polycythemia (Wang et al., 2019), and CMS (Zhang et al., 2021). Despite these studies, no information is available for plasma proteome level alterations of lowlanders during prolonged (months) stay at high altitude. Hence, we sought to investigate global plasma proteome alterations of lowlanders during a 3-month stay at 4,176 m as compared to 7 days stay at high altitude and sea level. In addition, we have also monitored physiological indices, proinflammatory cytokines, and lipid profiles. To the best of our knowledge, this is the first plasma proteomics investigation reporting molecular pathways perturbed during prolonged exposure to high altitude. We have further used biochemical estimation, ELISA studies, and Western blot analysis to validate our proteomics observations. Our present results indicate that acute exposure to high altitude induces dyslipidemia among lowlanders chronic exposure.

## Materials and Methods

### Study Groups

We studied 105 healthy, nonsmoking, male military volunteers (age: 22–37 years, height: 170 ± 4 cm, weight: 64 ± 3 kg) at sea level (Pathankot, India, altitude 331 m). All the volunteers are lowlanders-born, living at sea level, and have not been exposed to high altitude for the last 1 year. Subsequently, all the volunteers traveled to Leh (altitude 3,520 m) by road and stayed there for 7 days (high altitude–day seven group, (HA-D7 group; *n* = 55). Then, the volunteers spent 1 day reaching Fukche (4,176 m) and were stationed there for 3 months (high altitude–day 150 group (HA-D150 group; *n* = 40). All the volunteers followed the same diet regimen during the entire study period. The present study was performed according to the Declaration of Helsinki, and the experimental design and procedures for conducting the experiment were approved by the institutional ethics committee (IEC/DIPAS/B2/1). Informed written consent was obtained from all the participants and each volunteer was informed of the possible risk and discomforts involved in the study. During the whole study duration, regular medical examinations were performed to determine health status.

### Evaluation of Physiological Parameters

Physiological parameters including oxygen saturation (SpO_2_), heart rate (HR), systolic blood pressure (SBP), and diastolic blood pressure (DBP) were recorded at sea level and regularly at high altitudes. SpO_2_ was measured using a pulse oximeter (Smart Oxy Lite, BPL, India) from the right index finger as the average of three readings. Systolic blood pressure (SBP) and diastolic blood pressure (DBP) were recorded using a standard sphygmomanometer.

### Collection of Blood Plasma

Overnight fasting venous blood samples were drawn in EDTA vacutainer at Pathankot (sea level), Leh (HA-D7), and Fukche (HA-D150 groups). Plasma was separated by centrifugation at 1,500 x *g* for 15 min at 4°C and was stored at −80°C with mammalian protease inhibitor (P8340, Sigma-Aldrich) for further studies.

### TMT-Labeling and Plasma Protein Profiling

High abundant plasma proteins from each group (Sea level, HA-D7, and HA-D150) were depleted using High-Select™ Top14 Abundant Protein Depletion Resin (A36370, Thermo Scientific) according to the instructions of the manufacturer. An equal amount (100 μl) of the depleted sample (*n* = 8) was pooled together to comprise 800 μl of total volume for each group. Total protein content was determined with the ToPA Bradford Protein Assay kit (Cat No. K-0014, ITSI Biosciences, USA), and the consistency of protein profiles was checked using SDS-PAGE for each group. Subsequently, a 400 μg depleted protein sample was mixed with 75 μl of 8 M urea buffered in 1 M tetraethylammonium bromide (TEAB). The sample reduction was performed with 5 mM DL-dithiothreitol (DTT) for 30 min at 56°C, followed by alkylation with 11 mM iodoacetamide (IAM) for 15 min at room temperature in darkness. The sample was diluted by adding 200 mM TEAB and digested at a trypsin-to-protein mass ratio of 1:50 for the first digestion overnight and 1:100 for a 4-h digestion. Finally, each group of digested peptides was labeled with the TMT reagents following manufacturer protocols (Thermo Fisher Scientific, Torrance, CA, USA). Sample labeling was as follows: Sea level: 128, HA-D7: 129, and HA-D150: 130.

For multidimensional protein identification technology (MudPIT) analysis, all the labeled samples were combined and then fractionated by strong cation exchange (SCX). The fractions eluted at 50, 75, 150, 250, and 450 mM of ammonium acetate were collected. The samples were desalted using ZipTip, the desalted sample were dried in a speedvac, then resuspended in the appropriate mobile phase. For LC-MS/MS, the chromatography was performed with a Thermo EASY-nLCsystem. Peptides were eluted from the column using a linear acetonitrile gradient from 5 to 32% acetonitrile over 90 min followed by high and low organic washes for another 20 min coupled to Q Exactive™ mass spectrometer (Thermo Scientific) *via* a nanospray source with the spray voltage set to 2.0 kV and the ion transfer capillary set at 250°C. A data-dependent top 15 method was used where a full MS scan from m/z 350–1,600 was followed by MS/MS scans of the 15 most abundant ions. Each ion was subjected to Higher energy C trap dissociation (HCD) for fragmentation, peptide identification, and TMT reporter ion detection. Raw data files of each SCX fraction were searched against the most recent database for humans downloaded from UniProt using the MudPIT option in Proteome Discoverer 2.2 (Thermo Scientific) and the Sequest HT search algorithm. For protein identification results, only peptides identified with high confidence were used. For confidence, the Percolator algorithm was used for peptide spectrum match validation in database searches. The false discovery rate (FDR) threshold calculated in Proteome Discoverer Percolator with high confidence peptides (0.01) were used for protein identification.

### Pathway and Network Analysis

The identified proteins were analyzed according to GO terms for biological process, cellular component, and molecular function using the Reactome database (http://www.reactome). Pathway enrichment analysis was assessed using the Kyoto Encyclopedia of Genes and Genomes (KEGG) pathway database (http://www.genome.jp/kegg/ or http://www.kegg.jp/).

### Validation of Protein Levels by Immunoblotting

The protein levels in study groups were further validated with immunoblot analysis. Plasma containing 20 μg of protein was separated on 10% SDS-PAGE gels and transferred onto nitrocellulose membranes. The membranes were blocked in 5% BSA blocking buffer in PBS-0.1% Tween-20 (PBST) overnight at 4°C. Subsequently, the membranes were washed with PBST three times for 5 min each. It was followed by incubation with the Apo-B (PA5-86101, *Invitrogen*), Apo-CIII (701238, Thermo Fischer Scientific), and β-tubulin (MA516308, *Invitrogen*) for 2 h and secondary antibodies for 1.5 h at room temperature, respectively. The blots were washed again with PBST three times for 5 min each and were observed by adding chemiluminescent peroxidase substrate (Cat. No. 34095, ThermoFisher Scientific, USA) on UVP Biospectrum. Densitometry analysis was performed using Image J software.

### Lipid Profiling

Plasma lipid parameters including cholesterol, HDL, LDL, and triglycerides were analyzed on a Randox Monaco clinical chemistry analyzer (Randox Laboratories, Crumlin, UK).

### Evaluation of Inflammatory Cytokines

The plasma CRP level was evaluated using a human C-reactive protein ELISA Kit (E0829h, EIAab Science, Wuhan, CHINA) as per the instructions of the manufacturer. The levels of IL-6, TNFα, and ox-LDL were evaluated using Human IL-6 PicoKine™ ELISA Kit (EK0410, Boster Biologicals), Human TNFα ELISA Kit (950.090.096, Diaclone, Besancon Cedex, France), and human oxidized low density lipoprotein (OxLDL) ELISA Kit (E-EL-H0124, Elabscience), respectively, as per the recommendations of the manufacturer.

### Statistical Analysis

All the values were represented as mean ± SD. Statistical analysis was performed using ANOVA with Newman–Keuls *post-hoc* tests, and a *p*-value of < 0.05 was considered significant. All analysis was performed using GraphPad Prism software version 7.0 (GraphPad Software, California, USA). Pearson correlation analysis was performed for depicting the correlation between physiological and biochemical parameters.

## Results

### Evaluation of Physiological Parameters

Physiological parameters including SpO_2_, HR, SBP, DBP, and Hb were recorded for all the volunteers at sea level (lowlander) and also at HA (HA-D7 and HA-D150), and are represented in Table 1. Ascent to HA resulted in a decrease in SpO_2_ level for both HA-D7 (87.47 ± 2.85, *p* < 0.001) and HA-D150 (95.42 ± 0.78) as compared with sea level (98.99 ± 0.001). Comparison between HA-D7 and HA-D150 revealed an 8% increase (*p* < 0.001) of SpO_2_ during a long-term stay at HA. The HR increased upon HA stay, irrespective of duration, and the highest HR was observed for HA-D150 (83.14 ± 12.81 bpm, *p* < 0.001) as compared with both sea level (76.60 ± 8.26 bpm) and HA-D7 (77.51 ± 12.09 bpm). Similarly, SBP and DBP also increased upon HA ascent, and HA-D150 possessed the highest SBP (127.44 ± 12.99 mmHg, *p* < 0.001) and DBP (84.55 ± 9.16 mmHg, *p* < 0.001) as compared with both lowlander and HA-D7. HA exposure also resulted in increased hemoglobin for both HA-D7 (15.91 ± 0.62 g/dL, *p* < 0.01) and HA-D150 (15.30 ± 1.05, *p* < 0.05) as compared with lowlanders (14.07 ± 0.16 g/dL).

**Table 1:** Physiological parameters of volunteers at sea level (*n* = 105) who were subsequently exposed to HA for 7 days (HA-D7, *n* = 55) and 3 months (HA-D150, *n* = 40).

	**Sea level**	**HA-D7**	**HA-D150**
SpO_2_	98.99 ± 0.001	87.47 ± 2.855^***^	95.42 ± 0.785∧∧∧
HR (bpm)	76.60 ± 8.26	77.51 ± 12.09	83.14 ± 12.815∧∧∧
SBP (mmHg)	118.0 ± 8.97	120.68 ± 10.91	127.44 ± 12.995∧∧∧
DBP (mmHg)	78.40 ± 3.44	79.69 ± 8.65	84.55 ± 9.165∧∧∧
Hb (g/dL)	14.07 ± 0.16	15.91 ± 0.625^**^	15.30 ± 1.05^*^

* *represents p < 0.05 as compared to sea level*,

** *represents p < 0.01 as compared to sea level*,

*** *represents p < 0.001 as compared to sea level*,

∧∧∧ *represents p < 0.001 as compared to HA-D7*.

### Plasma Proteomics Study

**Figure 1:**
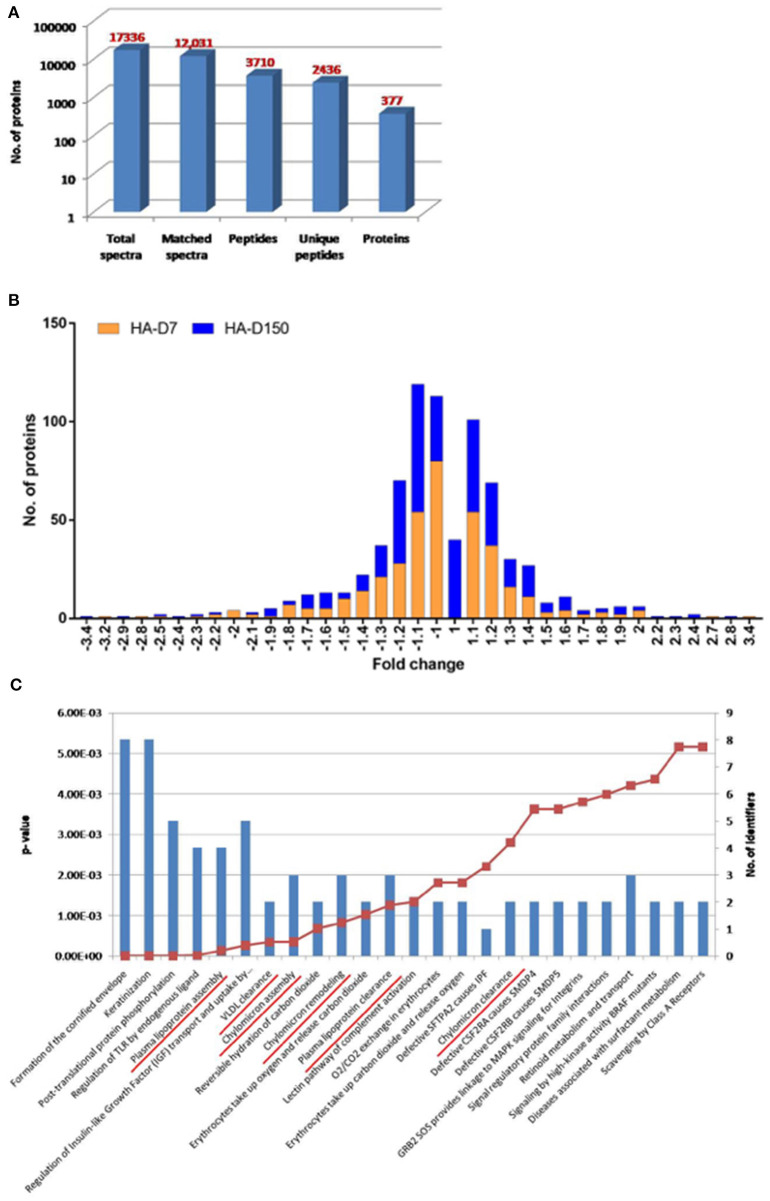
TMT based plasma proteomics analysis. **(A)** Bar graph representing spectra, peptides, and proteins identified from TMT-based LC-MS/MS. **(B)** The quantitative ratio histogram of quantitative proteins identified in the study groups (HA-D7 and HA-D150 with respect to sea level). **(C)** Top 25 significant pathways for the identified common upregulated proteins in HA-D7 and HA-D150 groups representing a number of identified proteins along with their *p*-value. Underlined signify identified pathways involved in lipoprotein metabolism.

Plasma proteomics studies were performed to identify global perturbations in plasma proteins and pathways during short- and long-term stay at HA. Plasma was first depleted for abundant proteins followed by TMT labeling and LC-MS/MS-based quantitative proteomics studies. A total of 17,336 peptides were identified in this experiment including low confidence peptides. However, with higher stringency (0.01% FDR), a total of 377 differentially abundant proteins were identified (Figure 1A; Supplementary Table 1). The frequency distribution of the 377 quantitative proteins, log2-transformed ratios fitted normality distribution (Figure 1B). For the HA-D7 group, a total of 75 proteins were found to be downregulated (< 0.8-fold) and 62 proteins were found to be upregulated (>1.2-fold), whereas 56 proteins were downregulated (<0.8-fold) and 79 proteins were upregulated (>1.2-fold) for HA-D150. Comparing both HA-D7 and HA-D150, we observed 32 downregulated proteins (<0.8-fold) and 27 upregulated proteins (>1.2-fold) common between short- and long term stay at high altitude (Supplementary Tables 2, 3).

Identified proteins were uploaded to a curated database “Reactome” (https://reactome.org) to identify underlying pathways altered during high altitude exposure. Interestingly, the top 25 significant pathways identified from common upregulated proteins at high altitude included plasma lipoprotein assembly, VLDL clearance, chylomicron assembly, chylomicron remodeling, plasma lipoprotein clearance, and chylomicron clearance (Figure 1C). In corroboration, we observed higher levels of apolipoproteins like APOB (1.32-fold), APOCI (1.36-fold), APOCIII (1.97-fold), APOCIV (1.34-fold), APOCIV + APOCII (1.15-fold), APOE (1.41-fold), and APOL (1.42-fold) for HA-D7. Similarly, we observed higher levels of Apo-AII (1.35-fold), Apo-B (1.34-fold), Apo-CI (1.39-fold), Apo-CIII (1.79-fold), Apo-CIV (1.17-fold), Apo-CIV + Apo-CII (1.20-fold), Apo-E (1.35-fold), and Apo-L (1.36-fold) for HA-D150. Comparing both the groups, we observed higher levels of carbonic anhydrase 1 (CA1, 2.30-fold) and carbonic anhydrase 2 (CA2, 2.36-fold) for HA-D150. We also observed a lower abundance of several cytoskeletal proteins like Profilin-1 (PROF-1), actin, cytoplasmic 2 (ACTG1), Talin-1 (TLN1), tubulin alpha chain, tubulin beta chain, myosin-9 (MYH9), and alpha-actinin-1 (ACTN1) in both the study groups (Supplementary Tables 1, 3).

### Validation of Plasma Proteomics Analysis

**Figure 2:**
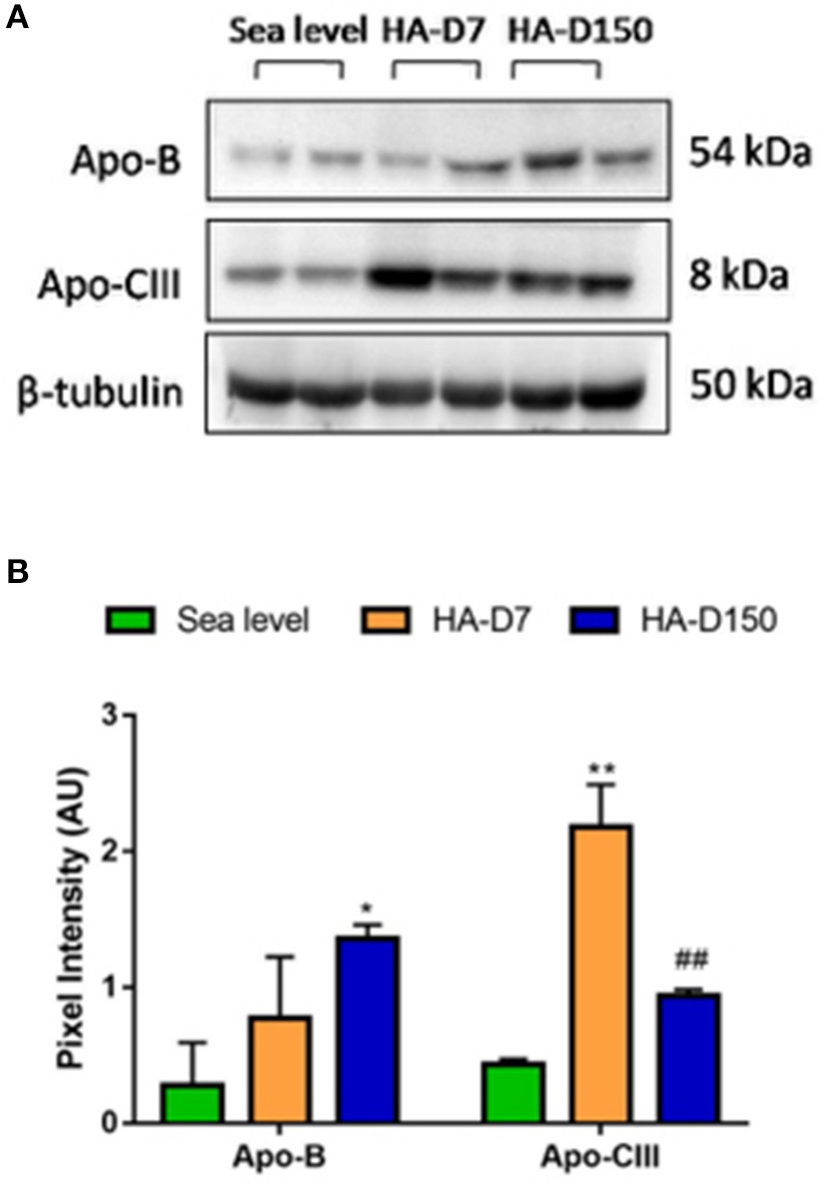
Validation of plasma proteomics data (*n* = 6). **(A)** Western blot analysis of Apo-B and Apo-CIII along with tubulin as a loading control. **(B)** Densitometry analysis of Apo-B and Apo-CIII with respect to tubulin. *represents *p* < 0.05, **represents *p* < 0.01 with respect to sea level and ^*##*^represents *p* < 0.01 with respect to HA-D7.

Validation of protein levels by western blot revealed that APOB levels were higher in HA-D7 (1.33-fold) and HA-D150 (2.36-fold) as compared with sea level. Similar higher levels of Apo-CIII (5.06-fold for HA-D7, p < 0.001, and 2.17-fold for HA-D150) were observed as compared to sea level (Figures 2A,B).

### Altered Lipid Profile During a Prolonged Stay at HA

To support the proteomics-based observations of altered apolipoproteins, lipid profiles of all the study groups were evaluated (Table 2). Elevated levels of plasma total cholesterol (225.63 ± 83.38 mg/dL, *p* < 0.05), LDL (135.1 ± 72.53 mg/dL, *p* < 0.05) and triglycerides (147.23 ± 76.74 mg/dL, *p* < 0.001) were observed for HAD-150 as compared with sea-level values. Concomitantly, higher plasma LDL level (135.1 ± 72.53mg/dL, *p* < 0.05) and lower HDL level (41.75 ± 4.98 mg/dL) was observed for HA-D150 as compared with sea-level values (112.17 ± 37.3 mg/dL and 47.19 ± 8.32 mg/dL, respectively). Consequently, the LDL/HDL ratio and total cholesterol/HDL ratio were elevated for HA-D150 (3.24 ± 14.58 and 5.40 ± 16.76, respectively) as compared to both HA-D7 and sea level. Moreover, the atherogenecity index of plasma (AIP) represented as log10 (TG/HDL-C) was also found to be highest in HA-D150 (Table 2).

**Table 2:** Assessment of lipid profile at sea level, 7 days, and 3 months (*n* = 8).

	**Sea level**	**HA-D7**	**HA-D150**
Triglycerides (mg/dL)	82.29 ± 31.04	105.62 ± 46.39	147.23 ± 76.745^***^
Total Cholesterol (mg/dL)	186.46 ± 50.08	175.21 ± 63.01	225.63 ± 83.385^*^ ∧
HDL (mg/dL)	47.19 ± 8.32	49.35 ± 14.90	41.75 ± 4.98
LDL (mg/dL)	112.17 ± 37.30	76.54 ± 25.255^*^	135.1 ± 72.535^*^ ∧
LDL/HDL ratio	2.38 ± 4.48	1.55 ± 1.69	3.24 ± 14.585^*^ ∧∧
Cholesterol/HDL ratio	3.95 ± 6.02	3.55 ± 4.23	5.40 ± 16.765^**^ ∧∧
Log_10_ (TG/ HDL-C), (AIP)	0.24	0.33	0.55

* *represents p < 0.05 as compared to sea level*,

** *represents p < 0.01 as compared to sea level*,

*** *represents p < 0.001 as compared to sea level*,

∧ *represents p < 0.05 as compared to HA-D7*,

∧∧ *represents p < 0.01 as compared to HA-D7. AIP indicates Atherogenecity Index of Plasma*.

### Evaluation of Inflammatory Markers

**Figure 3:**
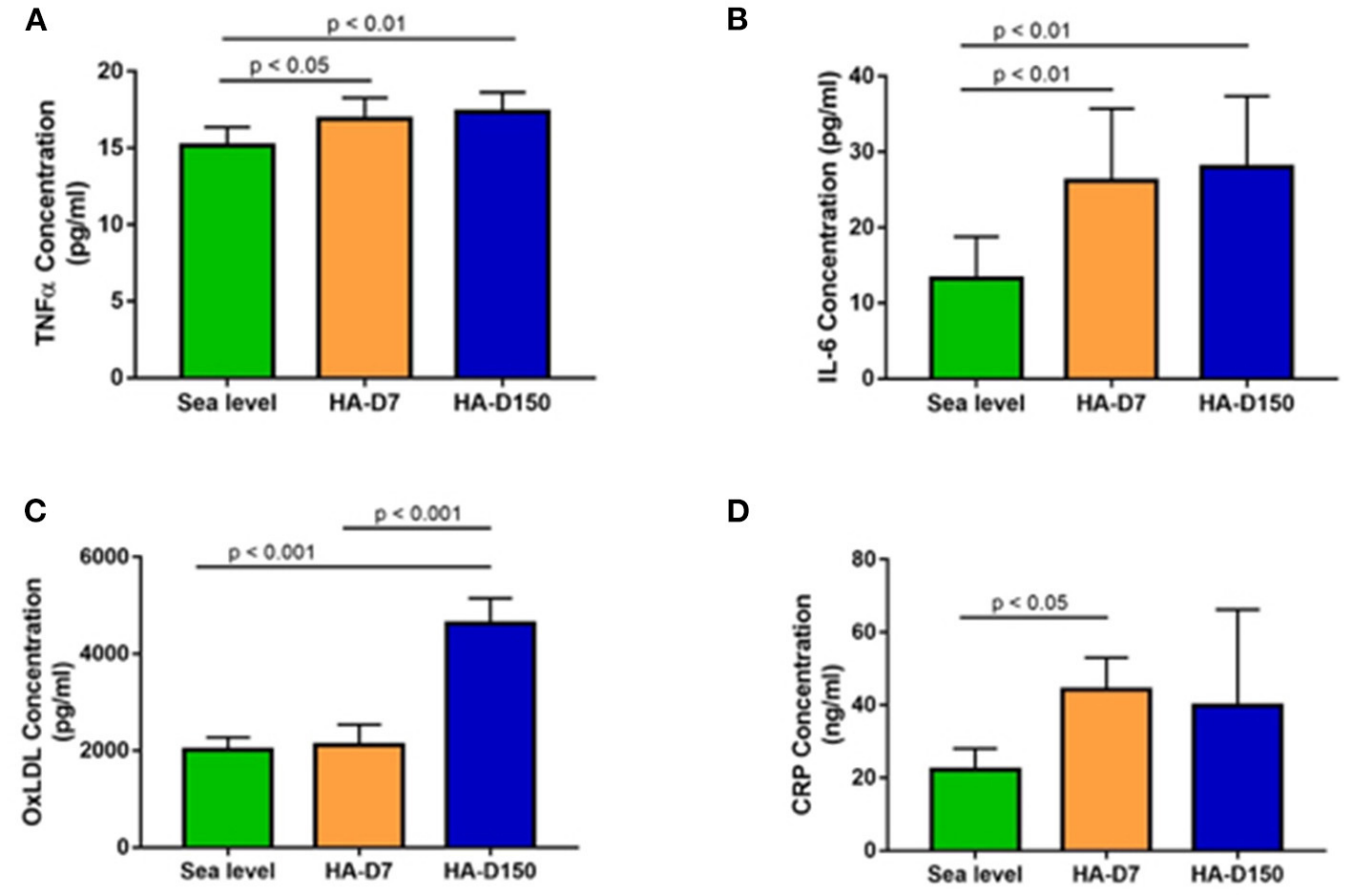
Assessment of inflammatory cytokines in the study groups (*n* = 8). ELISA-based estimation of **(A)** TNF-α, **(B)** IL-6, **(C)** ox-LDL, and **(D)** CRP for sea level, HA-D7, and HA-D150 groups.

We evaluated levels of inflammatory markers CRP, IL-6 and TNFα along with ox-LDL in all the study groups. We observed higher CRP levels in altitude exposed groups (1.99-fold for HA-D7, *p* < 0.05 and 1.79-fold for HA-D150) as compared to sea level values (Figure 3D). We observed similar higher levels of IL-6 and TNFα for HA-D7 (1.97-fold, *p* < 0.01 and 1.11-fold, *p* < 0.05) and HA-D150 (2.11-fold, *p* < 0.01 and 1.14-fold, *p* < 0.01) as compared to sea level values (Figures 3A,B). Interestingly, plasma level of ox-LDL was 2.29-fold (*p* < 0.001) and 2.18-fold (*p* < 0.001) higher in HA-D150 (4626 ± 520 pg/ml) as compared to sea level values (2018 ± 256 pg/ml) and HA-D7 (2119 ± 419.8 pg/ml) respectively (Figure 3C).

## Discussion

The present study reports the physiological and plasma proteome level changes for lowlanders during short- and long-term stay at high altitudes. Comparing the short-term and long-term plasma proteomes with sea level plasma proteome, the present study highlights activation of proatherogenic lipoproteins at high altitude that exacerbates during longer stay durations.

The arterial oxygen saturation level of lowlanders decreased upon acute exposure to high altitude (HA-D7) and increased significantly after long-term stay (HA-D150), though the levels were lower than sea level. This signifies healthy acclimatization to HA (Calbet et al., 2003; Soria et al., 2016) as severe and prolonged oxygen desaturation can lead to AMS (Mandolesi et al., 2014). As expected, the hemoglobin levels also increased significantly at high altitudes and persistently high levels were maintained irrespective of the duration of the stay at HA. Both the systolic and diastolic blood pressure along with heart rate increased upon HA exposure and was more pronounced during a long-term stay at HA. This is majorly due to massive activation of the sympathetic nervous system, contributed by noradrenaline despite enhanced hemoglobin levels and concomitant improved arterial oxygen content (Bartsch and Gibbs, 2007; Hainsworth et al., 2007).

Our TMT-based plasma proteomics studies identified a differential abundance of 377 proteins in HA-D7 and HA-D150 as compared to sea level. Using a cut-off of 0.8-fold for downregulation and 1.2-fold for upregulation, we identified 75 and 56 down-regulated proteins for HA-D7 and HA-D150 groups respectively. Similarly, we identified 62 and 79 upregulated proteins for HA-D7 and HA-D150 groups respectively. Bioinformatics analysis revealed alterations in several lipoprotein-associated pathways like plasma lipoprotein assembly, VLDL clearance, chylomicron assembly, plasma lipoprotein clearance, and chylomicron clearance. We observed higher levels of lipoproteins like APOB, APOCI, APOCIII, APOE, and APOL for both HA-D7 and HA-D150 in accordance with our previous studies (Gangwar et al., 2019). Interestingly, we observed higher levels of Apo-B for both HA-D7 and HA-D150 representing higher levels of low-density lipoprotein (LDL). Findings from several large studies indicate that elevated triglyceride (TG) levels along with increased levels of small dense LDL particles with concomitantly decreased levels of HDL cholesterol are often a component of atherogenic dyslipidemia (Reiner, 2017). We also observed similar high levels of APOCI, the endogenous inhibitor of cholesterol ester transfer protein (CETP) that limits the exchange of lipids has reportedly enhanced the risk of atherosclerosis (Westerterp et al., 2007). Our plasma proteomics and western blot studies identified higher APOCIII levels in high-altitude exposed groups. APOCIII is now recognized as a key regulator in severe hypertriglyceridemia due to its inhibition of lipoprotein lipase (LPL) and hepatic lipase. ApoCIII gain of function mutations are associated with atherosclerosis and coronary heart disease (CHD) and contribute to the development of hypertriglyceridemia, whereas loss of function mutations are associated with lower levels of plasma triglycerides and attenuation of vascular inflammatory processes (Pollin et al., 2008; Tg et al., 2014; Natarajan et al., 2015; Rocha et al., 2017).

Lipid profiling revealed higher levels of cholesterol, triglycerides, LDL, and lower levels of HDL were observed for HA-D150 as compared to HA-D7 and sea level groups (Table 2). It is noteworthy that sea-level values for total cholesterol, triglycerides (TGs), and LDL were within the physiological range, increased after high altitude exposure, and levels were further elevated during the prolonged stay. We also observed a moderate correlation of diastolic blood pressure (DBP) with LDL (R-squared = 0.46, *p* = 0.04) for HA-D7 group. Persistent hypoxia at high altitude induces HIF-1α that upregulates stearoyl-CoA desaturase (SCD)-1 in the sterol regulatory element-binding protein (SREBP)-1c pathway resulting in increased hepatic *de novo* TGs synthesis (Siques et al., 2020). In contrast, we observed the lowest HDL levels for HA-D150. High altitude exposure compromises HDL maturation and alters the levels of HDL associated proteins limiting its protective functions (Gangwar et al., 2019). Consequently, ratios of LDL/HDL, cholesterol/HDL, and AIP, potential risk factors for coronary artery disease (CAD) were elevated for HA-D150. Both animal and human studies have reported that hypoxia increases plasma triglycerides by decreasing tissue uptake (Barnholt et al., 2006; Siques et al., 2014). Epidemiological studies have reported a high prevalence of hypercholesterolemia and low HDL levels for indigenous high land native populations of Peru (Mohanna et al., 2006), Chile (Santos et al., 2001), and Tibet (Sherpa et al., 2011). These studies along with our current observation suggest that extended stay at high altitude results in dyslipidemia and elevates proatherogenic lipoprotein levels.

There is accumulating evidence that HA exposure is associated with an inflammatory response and associated endothelial activation/dysfunction (Hartmann et al., 2000; Bruno et al., 2014; Boos et al., 2016) that aggravates many forms of cardiovascular diseases (Riley and Gavin, 2017; Parati et al., 2018). Healthy young lowlanders with no preexisting risk factors develop massive infarct and ischemic stroke during a prolonged stay at HA (above 4,270 m) (Jha et al., 2002). Acclimatized lowlanders exhibit increased levels of coronary risk factors after 15–18 months stay at the Indian trans-Himalayan Ladakh region (altitude more than 3,500 m) (Dhar et al., 2018). Hence, we measured proinflammatory cytokines IL-6, CRP, TNFα, and oxidized LDL (oxLDL) levels in all three groups. The levels of IL-6 increased after altitude exposure (HA-D7) and levels were further increased after prolonged exposure (HA-D150). Studying cytokines after high altitude exposure, Hartmann et al. have reported increased IL-6 levels indicating considerable inflammation (Hartmann et al., 2000). Increased IL-6 level serves as an independent predictor of AMS (Boos et al., 2016) and plays a role in the pathogenesis of HAPE (Kubo et al., 1998). Similar higher levels of TNFα were observed after high altitude exposure and the highest levels were recorded for the HA-D150 group. Studying inflammatory cytokines in BAL fluid of HAPE patients, Kubo et al. have reported higher TNFα levels (Kubo et al., 1998) supporting our present observations. We also observed increased CRP levels for HA-D7 and the levels subsequently decreased for HA-D150 but remained higher than the sea level. Increased CRP levels have been reported for both acute (Hartmann et al., 2000; Gangwar et al., 2019) and prolonged high altitude exposure (Hu et al., 2016). Elevated levels of plasma CRP are associated with the risk of atherosclerotic events in general populations and show a predictive value even in terms of secondary prevention (Libby and Ridker, 2004; Calabro et al., 2009). Hence, we measured oxLDL levels that contribute to atherosclerotic plaque formation and progression by several mechanisms, including the induction of endothelial cell activation and dysfunction, macrophage foam cell formation, and smooth muscle cell migration and proliferation (Pirillo et al., 2013; Poznyak et al., 2020). We observed significantly higher oxLDL levels after a prolonged stay at high altitudes as compared to HA-D7 and sea level. Our present observations of elevated proinflammatory cytokines and oxLDL levels after prolonged exposure to high altitude indicate vascular inflammation leading to a proatherosclerotic state. A recent study evaluating long-term chronic intermittent hypobaric hypoxia-induced right ventricular hypertrophy has reported upregulation of lectin-like oxidized low-density lipoprotein receptor-1 (LOX-1), a major OxLDL receptor on endothelial cell surface supports our current observations (Pena et al., 2020b).

We identified CA1 and CA2 as the topmost upregulated proteins for HA-D150, on average 2-fold higher than the HA-D7 group. These two are a member of the CA family that reversibly catalyzes the hydration of CO_2_ to form ${\text{HCO}}_{3}^{-}$, which then rapidly binds to calcium ions to form calcium carbonate (Supuran, 2008). This well-known reaction is involved in a range of physiologic processes, ranging from CO_2_ metabolism to cell proliferation and glucose/lipid metabolism (Gilmour, 2010). It is important to note that acetazolamide and other related CA inhibitors have been effectively used for the prevention and treatment of AMS and remain the standard of care for this indication (Swenson, 2006; Nieto Estrada et al., 2017). While there exists no difference between red blood cell CA activity between lowlanders and native highlanders (Gamboa et al., 2000), increased activity has been reported in patients with obstructive sleep apnea (OSA) (Hoff et al., 2020). Inhibition of CA activity curtails inflammation and experimental hypertension (Hudalla et al., 2019) suggesting that CAs are involved in the inflammation and vascular calcification (Adeva-Andany et al., 2015; Yuan et al., 2019). Though our results do not provide any direct evidence for CA-mediated vascular inflammation and calcification, their profound upregulation and existence of a proatherogenic propensity during long-term stay at high altitude suggests a possible link between CA and high altitude-induced dyslipidemia.

The present study is limited by studying only plasma samples and blood parameters for understanding the body response to hypoxia, whereas at organ and tissue level the response is more complex. All global plasma proteomics studies are constrained by a high dynamic range of plasma proteins, and even after several high abundance protein depletion steps, it is not possible to detect very low abundance proteins that may be of critical importance. We have studied only male volunteers for 3 months in the present study for specific reasons. A vast number of studies have reported sexual dimorphism for cardiovascular response and disorders particularly due to the sex hormone estrogen (Hester et al., 2019; Horiuchi et al., 2019; Hou et al., 2019; Shen et al., 2020; Ndzie Noah et al., 2021). It is important to note that estrogen levels widely vary between both the sex (Khosla et al., 1998), and cardiac adaptive responses significantly vary between males and females during chronic hypoxia (Bohuslavova et al., 2010). Hence to minimize variations, women were consciously excluded from the present study. However, such proteomics studies with women and longer durations of high altitude stay (6 months or more) could provide additional molecular information.

In summary, the present study reports elevated levels of SpO_2_, HR, SBP, and DBP after long-term exposure to high altitude as compared to short-term exposure whereas the hemoglobin levels remained the same. Global plasma proteomics studies revealed upregulation of several apolipoproteins, and subsequent bioinformatics analysis revealed perturbation in assembly, remodeling, and clearance of plasma lipoproteins including VLDL and chylomicrons. In corroboration, we also observed higher TC, TGs, and LDL after a prolonged stay at high altitude indicating dyslipidemia. These observations were further supported by higher levels of inflammatory cytokines IL-6, TNFα, CRP as well as oxLDL. These cumulative results indicate persistent vascular inflammation after long-term exposure to high altitude leading to dyslipidemia and a proatherosclerotic condition.

## Data Availability Statement

The original contributions presented in the study are publicly available. This data can be found here: The mass spectrometry proteomics data have been deposited to the ProteomeXchange Consortium via the PRIDE partner repository with the dataset identifier PXD028070.

## Ethics Statement

The studies involving human participants were reviewed and approved by Ethics Committee of Defence Institute of Physiology and Allied Sciences (IEC/DIPAS/B2/1). The patients/participants provided their written informed consent to participate in this study.

## Author Contributions

NK designed the experiments and wrote the manuscript with inputs from P. P and VS performed the experiments. RM, KR, UP, and NK collected samples at sea level and high altitude as well as recorded physiological parameters. P, RV, and NK analyzed the data. All authors contributed to the article and approved the submitted version.

## Funding

This study was supported by Defence Research and Development Organisation (Project number DIP-263).

## Conflict of Interest

The authors declare that the research was conducted in the absence of any commercial or financial relationships that could be construed as a potential conflict of interest.

## Publisher's Note

All claims expressed in this article are solely those of the authors and do not necessarily represent those of their affiliated organizations, or those of the publisher, the editors and the reviewers. Any product that may be evaluated in this article, or claim that may be made by its manufacturer, is not guaranteed or endorsed by the publisher.
