# Supplementary material for: TMT-Based Plasma Proteomics Reveals Dyslipidemia Among Lowlanders During Prolonged Stay at High Altitudes
Source: Front Physiol. 2021 Oct 15;12:730601. doi: 10.3389/fphys.2021.730601 (PMC8554329; doi:10.3389/fphys.2021.730601)
Supplement: Supplementary file 1 [file Table_1.DOCX]

**Supplementary Table 1:** List of identified plasma proteins by TMT-labled LC-MS/MS analysis in HA-D7 and HA-D150 with respect to sea level.UniPort accession numbers, protein description along with fold-change for each group (HA-D7 and HA-D150) with respect to sea level values are mentioned.

| Accession | Description | HA-D7/ SL | HA-D150/ SL |
| --- | --- | --- | --- |
| C0JYY2 | Apolipoprotein B (Including Ag(X) antigen) OS=Homo sapiens GN=APOB PE=4 SV=1 | 0.989 | 1.117 |
| V9HWA9 | Epididymis secretory sperm binding protein Li 62p OS=Homo sapiens GN=HEL-S-62p PE=2 SV=1 | 1.094 | 1.004 |
| A0A0G2JPR0 | Complement C4-A OS=Homo sapiens GN=C4A PE=1 SV=1 | 0.931 | 1.01 |
| P02768 | Serum albumin OS=Homo sapiens GN=ALB PE=1 SV=2 | 0.91 | 0.759 |
| A0A140TA29 | Complement C4-B OS=Homo sapiens GN=C4B PE=1 SV=1 | 1.622 | 1.079 |
| P0C0L4 | Complement C4-A OS=Homo sapiens GN=C4A PE=1 SV=2 | 0.943 | 0.948 |
| Q59HB3 | Apolipoprotein B variant (Fragment) OS=Homo sapiens PE=2 SV=1 | 0.316 | 1.53 |
| Q7Z7Q0 | APOB protein OS=Homo sapiens GN=APOB PE=2 SV=1 | 1.327 | 1.346 |
| A8K5A4 | cDNA FLJ76826, highly similar to Homo sapiens ceruloplasmin (ferroxidase) (CP), mRNA OS=Homo sapiens PE=2 SV=1 | 0.964 | 0.958 |
| E9PFZ2 | Ceruloplasmin OS=Homo sapiens GN=CP PE=1 SV=1 | 1.609 | 0.089 |
| B4E1Z4 | cDNA FLJ55673, highly similar to Complement factor B (EC 3.4.21.47) OS=Homo sapiens PE=1 SV=1 | 1.041 | 1.076 |
| A0A024R962 | HCG40889, isoform CRA_b OS=Homo sapiens GN=hCG_40889 PE=4 SV=1 | 0.867 | 0.827 |
| A0A024R462 | Fibronectin 1, isoform CRA_n OS=Homo sapiens GN=FN1 PE=4 SV=1 | 1.279 | 1.365 |
| A8K5T0 | cDNA FLJ75416, highly similar to Homo sapiens complement factor H (CFH), mRNA OS=Homo sapiens PE=2 SV=1 | 0.66 | 0.598 |
| P01031 | Complement C5 OS=Homo sapiens GN=C5 PE=1 SV=4 | 0.983 | 0.901 |
| H6VRG1 | Keratin 1 OS=Homo sapiens GN=KRT1 PE=3 SV=1 | 1.918 | 1.452 |
| A0A024R3E3 | Apolipoprotein A-I, isoform CRA_a OS=Homo sapiens GN=APOA1 PE=3 SV=1 | 0.961 | 1.08 |
| P35908 | Keratin, type II cytoskeletal 2 epidermal OS=Homo sapiens GN=KRT2 PE=1 SV=2 | 1.159 | 1.759 |
| P06727 | Apolipoprotein A-IV OS=Homo sapiens GN=APOA4 PE=1 SV=3 | 0.872 | 0.99 |
| A0A024R944 | Serpin peptidase inhibitor, clade C (Antithrombin), member 1, isoform CRA_a OS=Homo sapiens GN=SERPINC1 PE=3 SV=1 | 1.231 | 1.01 |
| P00747 | Plasminogen OS=Homo sapiens GN=PLG PE=1 SV=2 | 0.808 | 0.838 |
| B7ZKJ8 | ITIH4 protein OS=Homo sapiens GN=ITIH4 PE=1 SV=1 | 0.88 | 0.933 |
| Q14624-2 | Isoform 2 of Inter-alpha-trypsin inhibitor heavy chain H4 OS=Homo sapiens GN=ITIH4 | 1.78 | 2.332 |
| P00734 | Prothrombin OS=Homo sapiens GN=F2 PE=1 SV=2 | 0.862 | 0.78 |
| D6RF35 | Vitamin D-binding protein OS=Homo sapiens GN=GC PE=1 SV=1 | 1.085 | 0.996 |
| P02774-3 | Isoform 3 of Vitamin D-binding protein OS=Homo sapiens GN=GC | 3.373 | 1.237 |
| B3KS79 | cDNA FLJ35730 fis, clone TESTI2003131, highly similar to ALPHA-1-ANTICHYMOTRYPSIN OS=Homo sapiens PE=2 SV=1 | 1.069 | 1.041 |
| D3DRR6 | Inter-alpha (Globulin) inhibitor H2, isoform CRA_a OS=Homo sapiens GN=ITIH2 PE=4 SV=1 | 1.108 | 0.946 |
| B7Z539 | cDNA FLJ56954, highly similar to Inter-alpha-trypsin inhibitor heavy chain H1 OS=Homo sapiens PE=2 SV=1 | 1.109 | 0.973 |
| B4E1C2 | Kininogen 1, isoform CRA_b OS=Homo sapiens GN=KNG1 PE=2 SV=1 | 0.959 | 0.917 |
| P13645 | Keratin, type I cytoskeletal 10 OS=Homo sapiens GN=KRT10 PE=1 SV=6 | 1.361 | 1.61 |
| A6XGL1 | Transthyretin OS=Homo sapiens PE=2 SV=1 | 1.04 | 0.978 |
| P01023 | Alpha-2-macroglobulin OS=Homo sapiens GN=A2M PE=1 SV=3 | 1.286 | 1.148 |
| P06396 | Gelsolin OS=Homo sapiens GN=GSN PE=1 SV=1 | 0.947 | 0.866 |
| D3DNU8 | Kininogen 1, isoform CRA_a OS=Homo sapiens GN=KNG1 PE=4 SV=1 | 0.766 | 0.886 |
| P35527 | Keratin, type I cytoskeletal 9 OS=Homo sapiens GN=KRT9 PE=1 SV=3 | 1.182 | 1.116 |
| P02790 | Hemopexin OS=Homo sapiens GN=HPX PE=1 SV=2 | 1.043 | 0.908 |
| P05155-3 | Isoform 3 of Plasma protease C1 inhibitor OS=Homo sapiens GN=SERPING1 | 1.015 | 0.902 |
| E9KL23 | Epididymis secretory sperm binding protein Li 44a OS=Homo sapiens GN=SERPINA1 PE=2 SV=1 | 0.797 | 0.718 |
| A0A024R6I7 | Alpha-1-antitrypsin OS=Homo sapiens GN=SERPINA1 PE=1 SV=1 | 0.895 | 0.943 |
| B7Z1F8 | cDNA FLJ53025, highly similar to Complement C4-B OS=Homo sapiens PE=2 SV=1 | 1.246 | 1.616 |
| P13671 | Complement component C6 OS=Homo sapiens GN=C6 PE=1 SV=3 | 1.003 | 0.967 |
| Q9UNU2 | Complement protein C4B frameshift mutant (Fragment) OS=Homo sapiens GN=C4B PE=4 SV=1 | 0.967 | 1.405 |
| P04003 | C4b-binding protein alpha chain OS=Homo sapiens GN=C4BPA PE=1 SV=2 | 0.961 | 0.966 |
| P25311 | Zinc-alpha-2-glycoprotein OS=Homo sapiens GN=AZGP1 PE=1 SV=2 | 1.065 | 1.006 |
| B4E1B2 | cDNA FLJ53691, highly similar to Serotransferrin OS=Homo sapiens PE=2 SV=1 | 0.975 | 0.804 |
| A0A1U9X8W4 | C2 OS=Homo sapiens PE=3 SV=1 | 0.905 | 1.015 |
| P02649 | Apolipoprotein E OS=Homo sapiens GN=APOE PE=1 SV=1 | 1.413 | 1.351 |
| P02671 | Fibrinogen alpha chain OS=Homo sapiens GN=FGA PE=1 SV=2 | 1.773 | 1.416 |
| P10909-2 | Isoform 2 of Clusterin OS=Homo sapiens GN=CLU | 1.079 | 1.011 |
| P43652 | Afamin OS=Homo sapiens GN=AFM PE=1 SV=1 | 0.835 | 0.845 |
| P05546 | Heparin cofactor 2 OS=Homo sapiens GN=SERPIND1 PE=1 SV=3 | 0.986 | 1.096 |
| D9YZU5 | Beta-globin OS=Homo sapiens GN=HBB PE=3 SV=1 | 0.956 | 6.44 |
| V9HWD8 | Epididymis secretory sperm binding protein Li 163pA OS=Homo sapiens GN=HEL-S-163pA PE=2 SV=1 | 0.976 | 0.966 |
| A8K2T4 | cDNA FLJ78207, highly similar to Human complement protein component C7 mRNA OS=Homo sapiens PE=2 SV=1 | 1.101 | 0.934 |
| P02749 | Beta-2-glycoprotein 1 OS=Homo sapiens GN=APOH PE=1 SV=3 | 0.701 | 0.756 |
| P04196 | Histidine-rich glycoprotein OS=Homo sapiens GN=HRG PE=1 SV=1 | 0.649 | 0.83 |
| B2R8I2 | cDNA, FLJ93914, highly similar to Homo sapiens histidine-rich glycoprotein (HRG), mRNA OS=Homo sapiens PE=2 SV=1 | 0.74 | 0.831 |
| P35858-2 | Isoform 2 of Insulin-like growth factor-binding protein complex acid labile subunit OS=Homo sapiens GN=IGFALS | 1.115 | 1.086 |
| P13647 | Keratin, type II cytoskeletal 5 OS=Homo sapiens GN=KRT5 PE=1 SV=3 | 1.106 | 1.271 |
| P05543 | Thyroxine-binding globulin OS=Homo sapiens GN=SERPINA7 PE=1 SV=2 | 1.134 | 1.066 |
| B4DPQ0 | Complement C1r subcomponent OS=Homo sapiens GN=C1R PE=1 SV=1 | 0.978 | 0.896 |
| P08697 | Alpha-2-antiplasmin OS=Homo sapiens GN=SERPINF2 PE=1 SV=3 | 1.064 | 0.963 |
| Q59EP2 | Angiotensinogen variant (Fragment) OS=Homo sapiens PE=2 SV=1 | 0.973 | 1.108 |
| A0A140VKF3 | Testis tissue sperm-binding protein Li 70n OS=Homo sapiens PE=2 SV=1 | 1.034 | 0.909 |
| A0A024R035 | Complement component 9, isoform CRA_a OS=Homo sapiens GN=C9 PE=4 SV=1 | 1.019 | 1.069 |
| A0A0S2Z428 | HCG2039812, isoform CRA_b (Fragment) OS=Homo sapiens GN=KRT6A PE=2 SV=1 | 1.5 | 1.408 |
| B2R815 | cDNA, FLJ93695, highly similar to Homo sapiens serpin peptidase inhibitor, clade A (alpha-1 antiproteinase, antitrypsin), member 4 (SERPINA4), mRNA OS=Homo sapiens PE=2 SV=1 | 0.909 | 0.922 |
| B7Z8B6 | cDNA FLJ54395, highly similar to Inter-alpha-trypsin inhibitor heavy chain H1 OS=Homo sapiens PE=2 SV=1 | 1.127 | 0.884 |
| B2R6W1 | cDNA, FLJ93143, highly similar to Homo sapiens complement component 7 (C7), mRNA OS=Homo sapiens PE=2 SV=1 | 0.679 | 1.559 |
| A8K9M5 | cDNA FLJ77947, highly similar to Human complement protein C8 beta subunit mRNA OS=Homo sapiens PE=2 SV=1 | 0.935 | 1.029 |
| A8K2N0 | cDNA FLJ77835, highly similar to Homo sapiens complement component 1, s subcomponent (C1S), transcript variant 2, mRNA OS=Homo sapiens PE=2 SV=1 | 0.993 | 0.931 |
| O75882 | Attractin OS=Homo sapiens GN=ATRN PE=1 SV=2 | 1.006 | 0.925 |
| P63261 | Actin, cytoplasmic 2 OS=Homo sapiens GN=ACTG1 PE=1 SV=1 | 0.472 | 0.44 |
| P02760 | Protein AMBP OS=Homo sapiens GN=AMBP PE=1 SV=1 | 0.708 | 0.706 |
| A0N071 | Delta globin OS=Homo sapiens GN=HBD PE=3 SV=1 | 0.853 | 3.988 |
| P02533 | Keratin, type I cytoskeletal 14 OS=Homo sapiens GN=KRT14 PE=1 SV=4 | 1.168 | 1.329 |
| H0YAC1 | Plasma kallikrein (Fragment) OS=Homo sapiens GN=KLKB1 PE=1 SV=1 | 0.989 | 0.929 |
| A0A0A0MRJ7 | Coagulation factor V OS=Homo sapiens GN=F5 PE=1 SV=1 | 0.927 | 0.959 |
| B2R9F2 | cDNA, FLJ94361, highly similar to Homo sapiens serine (or cysteine) proteinase inhibitor, clade A(alpha-1 antiproteinase, antitrypsin), member 6 (SERPINA6), mRNA OS=Homo sapiens PE=2 SV=1 | 0.944 | 0.996 |
| P00738 | Haptoglobin OS=Homo sapiens GN=HP PE=1 SV=1 | 0.95 | 1.232 |
| B7Z8Q2 | cDNA FLJ55606, highly similar to Alpha-2-HS-glycoprotein OS=Homo sapiens PE=2 SV=1 | 0.941 | 0.911 |
| P27169 | Serum paraoxonase/arylesterase 1 OS=Homo sapiens GN=PON1 PE=1 SV=3 | 1.028 | 0.952 |
| A0A0S2Z4L3 | Protein S isoform 2 (Fragment) OS=Homo sapiens GN=PROS1 PE=2 SV=1 | 0.98 | 0.977 |
| P00915 | Carbonic anhydrase 1 OS=Homo sapiens GN=CA1 PE=1 SV=2 | 1.253 | 2.301 |
| D9ZGG2 | Vitronectin OS=Homo sapiens GN=VTN PE=4 SV=1 | 1.094 | 1.01 |
| E7ETH0 | Complement factor I OS=Homo sapiens GN=CFI PE=1 SV=1 | 0.794 | 0.889 |
| Q06033 | Inter-alpha-trypsin inhibitor heavy chain H3 OS=Homo sapiens GN=ITIH3 PE=1 SV=2 | 1.031 | 0.894 |
| P22792 | Carboxypeptidase N subunit 2 OS=Homo sapiens GN=CPN2 PE=1 SV=3 | 1.021 | 1.027 |
| A0A024R930 | Proteoglycan 4, isoform CRA_a OS=Homo sapiens GN=PRG4 PE=4 SV=1 | 1.054 | 1.187 |
| B2R950 | cDNA, FLJ94213, highly similar to Homo sapiens pregnancy-zone protein (PZP), mRNA OS=Homo sapiens PE=2 SV=1 | 0.923 | 0.79 |
| A0A1K0GXZ1 | Globin C1 OS=Homo sapiens GN=GLNC1 PE=3 SV=1 | 0.837 | 4.082 |
| P07357 | Complement component C8 alpha chain OS=Homo sapiens GN=C8A PE=1 SV=2 | 0.935 | 0.929 |
| B2R6V9 | cDNA, FLJ93141, highly similar to Homo sapiens coagulation factor XIII, A1 polypeptide (F13A1), mRNA OS=Homo sapiens PE=2 SV=1 | 0.915 | 0.902 |
| P07360 | Complement component C8 gamma chain OS=Homo sapiens GN=C8G PE=1 SV=3 | 0.828 | 0.873 |
| B4DR57 | cDNA FLJ60818, highly similar to Complement C3 OS=Homo sapiens PE=2 SV=1 | 1.091 | 1.097 |
| P80108 | Phosphatidylinositol-glycan-specific phospholipase D OS=Homo sapiens GN=GPLD1 PE=1 SV=3 | 1.036 | 0.999 |
| P51884 | Lumican OS=Homo sapiens GN=LUM PE=1 SV=2 | 0.949 | 0.949 |
| Q6N095 | Uncharacterized protein OS=Homo sapiens GN=DKFZp686K03196 PE=1 SV=1 | 1.131 | 0.692 |
| Q6GMX6 | IGH@ protein OS=Homo sapiens GN=IGH@ PE=1 SV=1 | 1.104 | 0.702 |
| P08779 | Keratin, type I cytoskeletal 16 OS=Homo sapiens GN=KRT16 PE=1 SV=4 | 1.125 | 1.272 |
| B0YIW2 | Apolipoprotein C-III OS=Homo sapiens GN=APOC3 PE=1 SV=1 | 1.976 | 1.791 |
| Q86YZ3 | Hornerin OS=Homo sapiens GN=HRNR PE=1 SV=2 | 0.965 | 1.396 |
| C9JC84 | Fibrinogen gamma chain OS=Homo sapiens GN=FGG PE=1 SV=1 | 1.18 | 1.07 |
| P02750 | Leucine-rich alpha-2-glycoprotein OS=Homo sapiens GN=LRG1 PE=1 SV=2 | 0.935 | 1.174 |
| A8K1K1 | cDNA FLJ76342, highly similar to Homo sapiens carnosine dipeptidase 1 (metallopeptidase M20 family)(CNDP1), mRNA OS=Homo sapiens PE=2 SV=1 | 0.932 | 1.202 |
| A0A024R6N9 | Serpin peptidase inhibitor, clade A (Alpha-1 antiproteinase, antitrypsin), member 5, isoform CRA_a OS=Homo sapiens GN=SERPINA5 PE=3 SV=1 | 0.9 | 0.939 |
| P43251-2 | Isoform 2 of Biotinidase OS=Homo sapiens GN=BTD | 1.018 | 0.898 |
| P00742 | Coagulation factor X OS=Homo sapiens GN=F10 PE=1 SV=2 | 1.018 | 0.972 |
| A8K3K1 | cDNA FLJ78096, highly similar to Homo sapiens actin, alpha, cardiac muscle (ACTC), mRNA OS=Homo sapiens PE=2 SV=1 | 0.468 | 0.418 |
| Q6LAM1 | Heavy chain of factor I (Fragment) OS=Homo sapiens PE=2 SV=1 | 0.912 | 1.014 |
| P19652 | Alpha-1-acid glycoprotein 2 OS=Homo sapiens GN=ORM2 PE=1 SV=2 | 1.215 | 1.228 |
| P02743 | Serum amyloid P-component OS=Homo sapiens GN=APCS PE=1 SV=2 | 1.252 | 1.044 |
| Q03591 | Complement factor H-related protein 1 OS=Homo sapiens GN=CFHR1 PE=1 SV=2 | 0.912 | 1.133 |
| A0A0R7FJH5 | Coagulation factor XII OS=Homo sapiens GN=F12 PE=3 SV=1 | 0.947 | 0.809 |
| V9GYM3 | Apolipoprotein A-II OS=Homo sapiens GN=APOA2 PE=1 SV=1 | 0.99 | 1.355 |
| A0A024R1G8 | Apolipoprotein L, 1, isoform CRA_b OS=Homo sapiens GN=APOL1 PE=4 SV=1 | 1.423 | 1.367 |
| Q04695 | Keratin, type I cytoskeletal 17 OS=Homo sapiens GN=KRT17 PE=1 SV=2 | 1.397 | 0.892 |
| Q96PD5-2 | Isoform 2 of N-acetylmuramoyl-L-alanine amidase OS=Homo sapiens GN=PGLYRP2 | 0.959 | 0.979 |
| B2R888 | Monocyte differentiation antigen CD14 OS=Homo sapiens PE=2 SV=1 | 0.978 | 1.068 |
| A0A024R9Q1 | Thrombospondin 1, isoform CRA_a OS=Homo sapiens GN=THBS1 PE=4 SV=1 | 1.413 | 0.986 |
| P15169 | Carboxypeptidase N catalytic chain OS=Homo sapiens GN=CPN1 PE=1 SV=1 | 0.996 | 1.05 |
| P00739-2 | Isoform 2 of Haptoglobin-related protein OS=Homo sapiens GN=HPR | 1.012 | 1.228 |
| P01871-2 | Isoform 2 of Immunoglobulin heavy constant mu OS=Homo sapiens GN=IGHM | 0.806 | 0.637 |
| A0A0S2Z3Y1 | Lectin galactoside-binding soluble 3 binding protein isoform 1 (Fragment) OS=Homo sapiens GN=LGALS3BP PE=2 SV=1 | 1.01 | 1.026 |
| P06702 | Protein S100-A9 OS=Homo sapiens GN=S100A9 PE=1 SV=1 | 1.633 | 1.001 |
| P05160 | Coagulation factor XIII B chain OS=Homo sapiens GN=F13B PE=1 SV=3 | 1.015 | 1.017 |
| D3DNN4 | Carboxylic ester hydrolase OS=Homo sapiens GN=BCHE PE=3 SV=1 | 0.982 | 0.957 |
| P18428 | Lipopolysaccharide-binding protein OS=Homo sapiens GN=LBP PE=1 SV=3 | 0.933 | 1.11 |
| A0A024R0T8 | Apolipoprotein C-I, isoform CRA_a OS=Homo sapiens GN=APOC1 PE=4 SV=1 | 1.362 | 1.399 |
| P02746 | Complement C1q subcomponent subunit B OS=Homo sapiens GN=C1QB PE=1 SV=3 | 1.032 | 0.922 |
| Q6P5S8 | IGK@ protein OS=Homo sapiens GN=IGK@ PE=1 SV=1 | 0.814 | 0.63 |
| P23142 | Fibulin-1 OS=Homo sapiens GN=FBLN1 PE=1 SV=4 | 0.926 | 1.103 |
| P02763 | Alpha-1-acid glycoprotein 1 OS=Homo sapiens GN=ORM1 PE=1 SV=1 | 0.943 | 1.08 |
| K7ER74 | APOC4-APOC2 readthrough (NMD candidate) OS=Homo sapiens GN=APOC4-APOC2 PE=1 SV=1 | 1.156 | 1.204 |
| P02753 | Retinol-binding protein 4 OS=Homo sapiens GN=RBP4 PE=1 SV=3 | 0.837 | 0.977 |
| A0A024R2Q7 | C-type lectin domain family 3, member B, isoform CRA_a OS=Homo sapiens GN=CLEC3B PE=4 SV=1 | 0.819 | 0.791 |
| B2R582 | cDNA, FLJ92374, highly similar to Homo sapiens C-type lectin domain family 3, member B (CLEC3B), mRNA OS=Homo sapiens PE=2 SV=1 | 1.236 | 1.008 |
| Q96IY4 | Carboxypeptidase B2 OS=Homo sapiens GN=CPB2 PE=1 SV=2 | 1.023 | 1.03 |
| V9HVY1 | Epididymis secretory sperm binding protein Li 78p OS=Homo sapiens GN=HEL-S-78p PE=2 SV=1 | 1.239 | 1.024 |
| A0A096LPE2 | SAA2-SAA4 readthrough OS=Homo sapiens GN=SAA2-SAA4 PE=4 SV=1 | 1.047 | 1.256 |
| Q6GMV8 | Uncharacterized protein OS=Homo sapiens PE=2 SV=1 | 0.887 | 0.597 |
| Q7Z3Y8 | Keratin, type I cytoskeletal 27 OS=Homo sapiens GN=KRT27 PE=1 SV=2 | 0.866 | 1.009 |
| O95445 | Apolipoprotein M OS=Homo sapiens GN=APOM PE=1 SV=2 | 0.918 | 0.979 |
| G3V2W1 | Protein Z-dependent protease inhibitor OS=Homo sapiens GN=SERPINA10 PE=1 SV=1 | 0.954 | 1.027 |
| Q6GMX4 | IGL@ protein OS=Homo sapiens GN=IGL@ PE=1 SV=1 | 1.479 | 0.788 |
| P02741 | C-reactive protein OS=Homo sapiens GN=CRP PE=1 SV=1 | 1.096 | 4.49 |
| Q14520 | Hyaluronan-binding protein 2 OS=Homo sapiens GN=HABP2 PE=1 SV=1 | 1.076 | 1.069 |
| Q16610-4 | Isoform 4 of Extracellular matrix protein 1 OS=Homo sapiens GN=ECM1 | 0.932 | 0.975 |
| O75636 | Ficolin-3 OS=Homo sapiens GN=FCN3 PE=1 SV=2 | 0.919 | 1.04 |
| P20851 | C4b-binding protein beta chain OS=Homo sapiens GN=C4BPB PE=1 SV=1 | 0.994 | 1.055 |
| O00391 | Sulfhydryl oxidase 1 OS=Homo sapiens GN=QSOX1 PE=1 SV=3 | 0.952 | 0.944 |
| Q6UXB8 | Peptidase inhibitor 16 OS=Homo sapiens GN=PI16 PE=1 SV=1 | 0.842 | 0.857 |
| A0A140VK24 | Testicular secretory protein Li 24 OS=Homo sapiens PE=2 SV=1 | 1.158 | 1.079 |
| Q0IIN1 | Keratin 77 OS=Homo sapiens GN=KRT77 PE=1 SV=1 | 1.037 | 0.812 |
| Q6MZW0 | Uncharacterized protein DKFZp686J11235 (Fragment) OS=Homo sapiens GN=DKFZp686J11235 PE=1 SV=1 | 1.405 | 1.181 |
| C9JF17 | Apolipoprotein D (Fragment) OS=Homo sapiens GN=APOD PE=1 SV=1 | 0.992 | 1.06 |
| P05109 | Protein S100-A8 OS=Homo sapiens GN=S100A8 PE=1 SV=1 | 1.325 | 1.037 |
| Q6N092 | Uncharacterized protein DKFZp686K18196 (Fragment) OS=Homo sapiens GN=DKFZp686K18196 PE=2 SV=1 | 0.861 | 0.811 |
| P0DJI8 | Serum amyloid A-1 protein OS=Homo sapiens GN=SAA1 PE=1 SV=1 | 1.11 | 1.595 |
| B3KUE5 | Phospholipid transfer protein, isoform CRA_c OS=Homo sapiens GN=PLTP PE=2 SV=1 | 0.836 | 1.039 |
| P20930 | Filaggrin OS=Homo sapiens GN=FLG PE=1 SV=3 | 1.172 | 1.355 |
| Q5EBM2 | Uncharacterized protein OS=Homo sapiens PE=1 SV=1 | 1.281 | 0.769 |
| Q9UHG3 | Prenylcysteine oxidase 1 OS=Homo sapiens GN=PCYOX1 PE=1 SV=3 | 1.089 | 1.216 |
| V9HW12 | Epididymis secretory sperm binding protein Li 2a OS=Homo sapiens GN=HEL-S-2a PE=2 SV=1 | 1.102 | 2.794 |
| B2R701 | cDNA, FLJ93202, Homo sapiens protease inhibitor 16 (PI16), mRNA OS=Homo sapiens PE=2 SV=1 | 0.944 | 1.15 |
| G3XAK1 | Hepatocyte growth factor-like protein OS=Homo sapiens GN=MST1 PE=1 SV=1 | 0.967 | 0.913 |
| P31151 | Protein S100-A7 OS=Homo sapiens GN=S100A7 PE=1 SV=4 | 1.734 | 0.792 |
| P02747 | Complement C1q subcomponent subunit C OS=Homo sapiens GN=C1QC PE=1 SV=3 | 0.797 | 0.761 |
| D3DQX7 | Serum amyloid A protein OS=Homo sapiens GN=SAA1 PE=3 SV=1 | 1.121 | 1.25 |
| A0A1S5UZ07 | Talin-1 OS=Homo sapiens GN=TLN1 PE=2 SV=1 | 0.495 | 0.477 |
| Q9NZP8 | Complement C1r subcomponent-like protein OS=Homo sapiens GN=C1RL PE=1 SV=2 | 0.968 | 0.891 |
| D6RAR4 | Hepatocyte growth factor activator OS=Homo sapiens GN=HGFAC PE=1 SV=1 | 0.932 | 0.945 |
| P04040 | Catalase OS=Homo sapiens GN=CAT PE=1 SV=3 | 1.003 | 1.937 |
| U3PXP0 | Alpha globin chain (Fragment) OS=Homo sapiens GN=HBA2 PE=3 SV=1 | 0.545 | 1.551 |
| Q9UGM5 | Fetuin-B OS=Homo sapiens GN=FETUB PE=1 SV=2 | 1.155 | 1.047 |
| E2RVJ0 | Anion exchange protein OS=Homo sapiens GN=SLC4A1 PE=2 SV=1 | 0.703 | 1.492 |
| F2RM37 | Coagulation factor IX OS=Homo sapiens GN=F9 p22 PE=2 SV=1 | 1.004 | 1.011 |
| Q8TCZ8 | Apolipoprotein E (Fragment) OS=Homo sapiens GN=APOE PE=4 SV=1 | 0.842 | 0.59 |
| P21333 | Filamin-A OS=Homo sapiens GN=FLNA PE=1 SV=4 | 0.454 | 0.558 |
| P36980 | Complement factor H-related protein 2 OS=Homo sapiens GN=CFHR2 PE=1 SV=1 | 1.145 | 0.873 |
| Q6MZX7 | Uncharacterized protein DKFZp686M24218 OS=Homo sapiens GN=DKFZp686M24218 PE=2 SV=1 | 1.316 | 0.866 |
| P02775 | Platelet basic protein OS=Homo sapiens GN=PPBP PE=1 SV=3 | 1.731 | 0.851 |
| P02745 | Complement C1q subcomponent subunit A OS=Homo sapiens GN=C1QA PE=1 SV=2 | 1.165 | 0.906 |
| Q15582 | Transforming growth factor-beta-induced protein ig-h3 OS=Homo sapiens GN=TGFBI PE=1 SV=1 | 1.053 | 0.979 |
| P81605 | Dermcidin OS=Homo sapiens GN=DCD PE=1 SV=2 | 1.397 | 1.09 |
| B4DPQ3 | cDNA FLJ51034, highly similar to Vitamin K-dependent protein C (EC 3.4.21.69) OS=Homo sapiens PE=2 SV=1 | 1.087 | 1.085 |
| Q9NQ79 | Cartilage acidic protein 1 OS=Homo sapiens GN=CRTAC1 PE=1 SV=2 | 0.85 | 0.824 |
| P22352 | Glutathione peroxidase 3 OS=Homo sapiens GN=GPX3 PE=1 SV=2 | 1.067 | 1.078 |
| Q08554 | Desmocollin-1 OS=Homo sapiens GN=DSC1 PE=1 SV=2 | 1.248 | 1.265 |
| V9HWB4 | Epididymis secretory sperm binding protein Li 89n OS=Homo sapiens GN=HEL-S-89n PE=2 SV=1 | 1.053 | 1.045 |
| P03951 | Coagulation factor XI OS=Homo sapiens GN=F11 PE=1 SV=1 | 0.69 | 0.795 |
| S6AWF4 | IgG L chain OS=Homo sapiens PE=2 SV=1 | 1.392 | 0.849 |
| B2R5G8 | Serum amyloid A protein OS=Homo sapiens PE=2 SV=1 | 0.885 | 0.946 |
| Q7Z3Z0 | Keratin, type I cytoskeletal 25 OS=Homo sapiens GN=KRT25 PE=1 SV=1 | 0.905 | 1.103 |
| E0D851 | Platelet glycoprotein Ib alpha OS=Homo sapiens GN=GP1BA PE=4 SV=1 | 0.889 | 0.873 |
| P29508 | Serpin B3 OS=Homo sapiens GN=SERPINB3 PE=1 SV=2 | 1.309 | 0.751 |
| Q1HP67 | Lipoprotein, Lp(A) OS=Homo sapiens GN=LPA PE=3 SV=1 | 0.646 | 0.914 |
| P00918 | Carbonic anhydrase 2 OS=Homo sapiens GN=CA2 PE=1 SV=2 | 1.304 | 2.366 |
| P04275 | von Willebrand factor OS=Homo sapiens GN=VWF PE=1 SV=4 | 1.361 | 1.193 |
| Q6EMK4 | Vasorin OS=Homo sapiens GN=VASN PE=1 SV=1 | 1.023 | 0.966 |
| P17936-2 | Isoform 2 of Insulin-like growth factor-binding protein 3 OS=Homo sapiens GN=IGFBP3 | 0.932 | 0.999 |
| A8K6Q8 | cDNA FLJ75881, highly similar to Homo sapiens transferrin receptor (p90, CD71) (TFRC), mRNA OS=Homo sapiens PE=2 SV=1 | 1.309 | 0.98 |
| P14151-2 | Isoform 2 of L-selectin OS=Homo sapiens GN=SELL | 1.165 | 0.903 |
| P11226 | Mannose-binding protein C OS=Homo sapiens GN=MBL2 PE=1 SV=2 | 1.243 | 1.345 |
| A0M8Q6 | Immunoglobulin lambda constant 7 OS=Homo sapiens GN=IGLC7 PE=1 SV=3 | 0.917 | 0.814 |
| A6XNE2 | Complement factor D preproprotein OS=Homo sapiens PE=2 SV=1 | 1.067 | 1.004 |
| P04278 | Sex hormone-binding globulin OS=Homo sapiens GN=SHBG PE=1 SV=2 | 0.804 | 0.918 |
| Q5D862 | Filaggrin-2 OS=Homo sapiens GN=FLG2 PE=1 SV=1 | 1.994 | 2.153 |
| A0A0S2Z4I5 | Complement factor properdin isoform 1 (Fragment) OS=Homo sapiens GN=CFP PE=2 SV=1 | 0.593 | 0.605 |
| Q8N1N4 | Keratin, type II cytoskeletal 78 OS=Homo sapiens GN=KRT78 PE=1 SV=2 | 1.074 | 1.251 |
| P30043 | Flavin reductase (NADPH) OS=Homo sapiens GN=BLVRB PE=1 SV=3 | 1.125 | 1.596 |
| P49908 | Selenoprotein P OS=Homo sapiens GN=SELENOP PE=1 SV=3 | 0.879 | 0.928 |
| Q9Y6R7 | IgGFc-binding protein OS=Homo sapiens GN=FCGBP PE=1 SV=3 | 1.115 | 0.937 |
| A0A024R1X8 | Junction plakoglobin, isoform CRA_a OS=Homo sapiens GN=JUP PE=4 SV=1 | 0.752 | 1.172 |
| O00533-2 | Isoform 2 of Neural cell adhesion molecule L1-like protein OS=Homo sapiens GN=CHL1 | 1.176 | 1.242 |
| Q59EA3 | Cadherin 5, type 2 preproprotein variant (Fragment) OS=Homo sapiens PE=2 SV=1 | 1.086 | 0.926 |
| Q9H804 | cDNA FLJ14022 fis, clone HEMBA1003538, weakly similar to COMPLEMENT C1R COMPONENT (EC 3.4.21.41) OS=Homo sapiens PE=2 SV=1 | 0.645 | 0.81 |
| A0A0X9USM3 | GCT-A4 heavy chain variable region (Fragment) OS=Homo sapiens PE=2 SV=1 | 1.114 | 0.807 |
| P04075-2 | Isoform 2 of Fructose-bisphosphate aldolase A OS=Homo sapiens GN=ALDOA | 0.689 | 0.902 |
| A5YAK2 | Apolipoprotein C-IV OS=Homo sapiens GN=APOC4 PE=2 SV=1 | 1.348 | 1.175 |
| P67936-2 | Isoform 2 of Tropomyosin alpha-4 chain OS=Homo sapiens GN=TPM4 | 0.524 | 0.546 |
| Q99784 | Noelin OS=Homo sapiens GN=OLFM1 PE=1 SV=4 | 1.019 | 0.934 |
| P04406 | Glyceraldehyde-3-phosphate dehydrogenase OS=Homo sapiens GN=GAPDH PE=1 SV=3 | 0.757 | 0.931 |
| E7EX29 | 14-3-3 protein zeta/delta (Fragment) OS=Homo sapiens GN=YWHAZ PE=1 SV=1 | 0.607 | 0.717 |
| A0A024RDE1 | SPARC-like 1 (Mast9, hevin), isoform CRA_a OS=Homo sapiens GN=SPARCL1 PE=4 SV=1 | 0.854 | 0.915 |
| Q59E93 | Aminopeptidase (Fragment) OS=Homo sapiens PE=2 SV=1 | 1.155 | 0.917 |
| Q02985 | Complement factor H-related protein 3 OS=Homo sapiens GN=CFHR3 PE=1 SV=2 | 0.792 | 1.102 |
| B4E0X1 | Beta-2-microglobulin OS=Homo sapiens PE=2 SV=1 | 0.693 | 0.781 |
| V9HWK2 | Epididymis luminal protein 114 OS=Homo sapiens GN=HEL114 PE=2 SV=1 | 0.6 | 0.608 |
| A0A193CHQ9 | 10E8 heavy chain variable region (Fragment) OS=Homo sapiens PE=2 SV=1 | 1.349 | 1.082 |
| A0A075B6S2 | Immunoglobulin kappa variable 2D-29 OS=Homo sapiens GN=IGKV2D-29 PE=3 SV=1 | 1.078 | 0.666 |
| A2KBB9 | Anti-(ED-B) scFV (Fragment) OS=Homo sapiens PE=2 SV=1 | 1.031 | 1.335 |
| A0A0K0K1J1 | Cystatin OS=Homo sapiens GN=HEL-S-2 PE=2 SV=1 | 1.55 | 1.053 |
| V9H1C1 | Gelsolin exon 4 (Fragment) OS=Homo sapiens PE=4 SV=1 | 0.992 | 0.949 |
| P60174 | Triosephosphate isomerase OS=Homo sapiens GN=TPI1 PE=1 SV=3 | 0.651 | 1.168 |
| P15924 | Desmoplakin OS=Homo sapiens GN=DSP PE=1 SV=3 | 1.218 | 1.17 |
| A0A1B1CYC5 | Vitamin D binding protein (Fragment) OS=Homo sapiens GN=Gc PE=4 SV=1 | 1.977 | 1.371 |
| P10720 | Platelet factor 4 variant OS=Homo sapiens GN=PF4V1 PE=1 SV=1 | 0.986 | 0.835 |
| Q6UWP8 | Suprabasin OS=Homo sapiens GN=SBSN PE=1 SV=2 | 0.756 | 1.133 |
| P35579 | Myosin-9 OS=Homo sapiens GN=MYH9 PE=1 SV=4 | 0.562 | 0.521 |
| Q15166 | Serum paraoxonase/lactonase 3 OS=Homo sapiens GN=PON3 PE=1 SV=3 | 0.952 | 1.217 |
| B2R7D2 | cDNA, FLJ93389, highly similar to Homo sapiens multiple inositol polyphosphate histidine phosphatase, 1 (MINPP1), mRNA OS=Homo sapiens PE=2 SV=1 | 1.127 | 1.196 |
| P09172 | Dopamine beta-hydroxylase OS=Homo sapiens GN=DBH PE=1 SV=3 | 1.195 | 1.136 |
| P22891-2 | Isoform 2 of Vitamin K-dependent protein Z OS=Homo sapiens GN=PROZ | 1.05 | 1.102 |
| Q3SY84 | Keratin, type II cytoskeletal 71 OS=Homo sapiens GN=KRT71 PE=1 SV=3 | 0.95 | 0.846 |
| A0A024RDT4 | Lymphocyte cytosolic protein 1 (L-plastin), isoform CRA_a OS=Homo sapiens GN=LCP1 PE=4 SV=1 | 0.976 | 0.892 |
| Q14403 | Gamma-G globin (Fragment) OS=Homo sapiens PE=2 SV=1 | 0.808 | 1.402 |
| P61626 | Lysozyme C OS=Homo sapiens GN=LYZ PE=1 SV=1 | 1.236 | 1.039 |
| B5BU24 | 14-3-3 protein beta/alpha OS=Homo sapiens GN=YWHAB PE=2 SV=1 | 0.502 | 0.689 |
| P37802-2 | Isoform 2 of Transgelin-2 OS=Homo sapiens GN=TAGLN2 | 1.159 | 0.905 |
| P07737 | Profilin-1 OS=Homo sapiens GN=PFN1 PE=1 SV=2 | 0.351 | 0.349 |
| P12814-4 | Isoform 4 of Alpha-actinin-1 OS=Homo sapiens GN=ACTN1 | 0.652 | 0.713 |
| P34096 | Ribonuclease 4 OS=Homo sapiens GN=RNASE4 PE=1 SV=3 | 0.741 | 1.124 |
| P48740 | Mannan-binding lectin serine protease 1 OS=Homo sapiens GN=MASP1 PE=1 SV=3 | 1.179 | 1.064 |
| Q53GZ6 | Heat shock 70kDa protein 8 isoform 1 variant (Fragment) OS=Homo sapiens PE=2 SV=1 | 0.743 | 0.831 |
| A8K3I0 | cDNA FLJ78437, highly similar to Homo sapiens cartilage oligomeric matrix protein (COMP), mRNA OS=Homo sapiens PE=2 SV=1 | 0.459 | 1.096 |
| V9HWI3 | Cathepsin D (Lysosomal aspartyl peptidase), isoform CRA_a OS=Homo sapiens GN=HEL-S-130P PE=2 SV=1 | 0.8 | 0.832 |
| A8K335 | cDNA FLJ76254, highly similar to Homo sapiens gamma-glutamyl hydrolase (GGH), mRNA OS=Homo sapiens PE=2 SV=1 | 1.139 | 1.043 |
| P23470 | Receptor-type tyrosine-protein phosphatase gamma OS=Homo sapiens GN=PTPRG PE=1 SV=4 | 0.95 | 0.898 |
| P16070 | CD44 antigen OS=Homo sapiens GN=CD44 PE=1 SV=3 | 0.836 | 0.813 |
| Q13201 | Multimerin-1 OS=Homo sapiens GN=MMRN1 PE=1 SV=3 | 0.691 | 0.861 |
| A2NB46 | Cold agglutinin FS-2 L-chain (Fragment) OS=Homo sapiens PE=2 SV=1 | 1.011 | 1.239 |
| P22105-4 | Isoform 5 of Tenascin-X OS=Homo sapiens GN=TNXB | 1.089 | 1.057 |
| P62979 | Ubiquitin-40S ribosomal protein S27a OS=Homo sapiens GN=RPS27A PE=1 SV=2 | 1.043 | 1.369 |
| A8K6C1 | cDNA FLJ76868, highly similar to Homo sapiens cholesteryl ester transfer protein, plasma (CETP), mRNA OS=Homo sapiens PE=2 SV=1 | 0.766 | 1.091 |
| Q9UNN8 | Endothelial protein C receptor OS=Homo sapiens GN=PROCR PE=1 SV=1 | 0.82 | 1.203 |
| P48740-2 | Isoform 2 of Mannan-binding lectin serine protease 1 OS=Homo sapiens GN=MASP1 | 0.747 | 0.848 |
| B7Z1K5 | Tubulin alpha chain OS=Homo sapiens PE=2 SV=1 | 0.51 | 0.463 |
| P03950 | Angiogenin OS=Homo sapiens GN=ANG PE=1 SV=1 | 1.005 | 0.878 |
| P98160 | Basement membrane-specific heparan sulfate proteoglycan core protein OS=Homo sapiens GN=HSPG2 PE=1 SV=4 | 0.801 | 0.803 |
| A0A024RD39 | Platelet-activating factor acetylhydrolase OS=Homo sapiens GN=PLA2G7 PE=4 SV=1 | 1.114 | 1.136 |
| G9K388 | YWHAE/FAM22A fusion protein (Fragment) OS=Homo sapiens GN=YWHAE/FAM22A fusion PE=2 SV=1 | 0.89 | 0.921 |
| P26038 | Moesin OS=Homo sapiens GN=MSN PE=1 SV=3 | 0.619 | 0.953 |
| B2R773 | cDNA, FLJ93312, highly similar to Homo sapiens adipose most abundant gene transcript 1 (APM1), mRNA OS=Homo sapiens PE=2 SV=1 | 0.983 | 1.159 |
| O43866 | CD5 antigen-like OS=Homo sapiens GN=CD5L PE=1 SV=1 | 0.838 | 0.663 |
| P00338-3 | Isoform 3 of L-lactate dehydrogenase A chain OS=Homo sapiens GN=LDHA | 0.684 | 0.617 |
| V9HWC7 | Epididymis secretory sperm binding protein Li 128m OS=Homo sapiens GN=HEL-S-128m PE=2 SV=1 | 0.972 | 1.472 |
| B2RC09 | cDNA, FLJ95794, highly similar to Homo sapiens apolipoprotein F (APOF), mRNA OS=Homo sapiens PE=2 SV=1 | 0.611 | 0.888 |
| Q9Y5Y7 | Lymphatic vessel endothelial hyaluronic acid receptor 1 OS=Homo sapiens GN=LYVE1 PE=1 SV=2 | 1.121 | 1.016 |
| B2RAN2 | cDNA, FLJ95014, highly similar to Homo sapiens vanin 1 (VNN1), mRNA OS=Homo sapiens PE=2 SV=1 | 0.977 | 1.205 |
| P62805 | Histone H4 OS=Homo sapiens GN=HIST1H4A PE=1 SV=2 | 1.342 | 0.925 |
| A0A0C4DG49 | Poliovirus receptor OS=Homo sapiens GN=PVR PE=1 SV=1 | 1.121 | 0.957 |
| J3KPA1 | Cysteine-rich secretory protein 3 OS=Homo sapiens GN=CRISP3 PE=1 SV=1 | 1.012 | 0.869 |
| P08514 | Integrin alpha-IIb OS=Homo sapiens GN=ITGA2B PE=1 SV=3 | 0.603 | 0.532 |
| Q9H8L6 | Multimerin-2 OS=Homo sapiens GN=MMRN2 PE=1 SV=2 | 1.14 | 1.024 |
| C9IZP8 | Complement C1s subcomponent (Fragment) OS=Homo sapiens GN=C1S PE=1 SV=1 | 0.955 | 0.988 |
| B2R4P2 | cDNA, FLJ92164, highly similar to Homo sapiens peroxiredoxin 1 (PRDX1), mRNA OS=Homo sapiens PE=2 SV=1 | 1.239 | 1.655 |
| E7EMB3 | Calmodulin-2 OS=Homo sapiens GN=CALM2 PE=1 SV=1 | 0.741 | 0.636 |
| P08709 | Coagulation factor VII OS=Homo sapiens GN=F7 PE=1 SV=1 | 1.173 | 1.039 |
| B2RBW9 | cDNA, FLJ95746, highly similar to Homo sapiens inhibin, beta C (INHBC), mRNA OS=Homo sapiens PE=2 SV=1 | 1.172 | 1.015 |
| P24592 | Insulin-like growth factor-binding protein 6 OS=Homo sapiens GN=IGFBP6 PE=1 SV=1 | 1.056 | 0.927 |
| Q86U17 | Serpin A11 OS=Homo sapiens GN=SERPINA11 PE=2 SV=2 | 0.875 | 0.894 |
| Q5U077 | L-lactate dehydrogenase OS=Homo sapiens GN=LDHB PE=2 SV=1 | 0.888 | 0.867 |
| A0A024R782 | Phosphoglycerate mutase OS=Homo sapiens GN=BPGM PE=2 SV=1 | 0.986 | 1.969 |
| B2R932 | cDNA, FLJ94187, highly similar to Homo sapiens CD99 antigen (CD99), mRNA OS=Homo sapiens PE=2 SV=1 | 0.559 | 0.94 |
| Q6UX71 | Plexin domain-containing protein 2 OS=Homo sapiens GN=PLXDC2 PE=1 SV=1 | 1.036 | 1.947 |
| Q13822-2 | Isoform 2 of Ectonucleotidepyrophosphatase/phosphodiesterase family member 2 OS=Homo sapiens GN=ENPP2 | 1.074 | 0.957 |
| P06703 | Protein S100-A6 OS=Homo sapiens GN=S100A6 PE=1 SV=1 | 0.893 | 1.855 |
| A8KAJ3 | cDNA FLJ77823, highly similar to Homo sapiens EGF-containing fibulin-like extracellular matrix protein 1, transcript variant 3, mRNA OS=Homo sapiens PE=2 SV=1 | 1.07 | 0.817 |
| A5D6W6 | Fat storage-inducing transmembrane protein 1 OS=Homo sapiens GN=FITM1 PE=2 SV=1 | 0.788 | 1.16 |
| Q6ZVX7 | F-box only protein 50 OS=Homo sapiens GN=NCCRP1 PE=1 SV=1 | 1.274 | 1.604 |
| D3DQH8 | Secreted protein, acidic, cysteine-rich (Osteonectin), isoform CRA_a OS=Homo sapiens GN=SPARC PE=4 SV=1 | 1.34 | 0.849 |
| A0A087WVC6 | Tyrosine-protein phosphatase OS=Homo sapiens GN=PTPRJ PE=1 SV=1 | 0.713 | 0.75 |
| P24593 | Insulin-like growth factor-binding protein 5 OS=Homo sapiens GN=IGFBP5 PE=1 SV=1 | 1.023 | 1.132 |
| A8K061 | cDNA FLJ77880, highly similar to Homo sapiens angiopoietin-like 3, mRNA OS=Homo sapiens PE=2 SV=1 | 0.491 | 0.514 |
| P12273 | Prolactin-inducible protein OS=Homo sapiens GN=PIP PE=1 SV=1 | 0.687 | 0.764 |
| M9MML0 | Low affinity immunoglobulin gamma Fc region receptor III-A OS=Homo sapiens GN=FCGR3A PE=4 SV=2 | 1.173 | 1.084 |
| Q13103 | Secreted phosphoprotein 24 OS=Homo sapiens GN=SPP2 PE=1 SV=1 | 0.938 | 0.842 |
| A0A0X9V9B3 | MS-F1 light chain variable region (Fragment) OS=Homo sapiens PE=2 SV=1 | 1.056 | 0.601 |
| K4DIA0 | ICOS ligand OS=Homo sapiens GN=ICOSLG PE=1 SV=1 | 1.072 | 0.899 |
| Q16706 | Alpha-mannosidase 2 OS=Homo sapiens GN=MAN2A1 PE=1 SV=2 | 0.793 | 0.911 |
| R4GMU1 | GDH/6PGL endoplasmic bifunctional protein OS=Homo sapiens GN=H6PD PE=1 SV=1 | 1.146 | 1.226 |
| Q59ED3 | Intercellular adhesion molecule 2 variant (Fragment) OS=Homo sapiens PE=2 SV=1 | 1.186 | 1.005 |
| A0N7J6 | REV25-2 (Fragment) OS=Homo sapiens PE=2 SV=1 | 0.942 | 0.747 |
| P18065 | Insulin-like growth factor-binding protein 2 OS=Homo sapiens GN=IGFBP2 PE=1 SV=2 | 1.036 | 0.964 |
| Q59GR7 | Uncharacterized protein (Fragment) OS=Homo sapiens PE=4 SV=1 | 0.954 | 0.4 |
| P25786-2 | Isoform Long of Proteasome subunit alpha type-1 OS=Homo sapiens GN=PSMA1 | 0.791 | 1.159 |
| Q9NPH3-5 | Isoform 4 of Interleukin-1 receptor accessory protein OS=Homo sapiens GN=IL1RAP | 1.183 | 0.809 |
| P12111 | Collagen alpha-3(VI) chain OS=Homo sapiens GN=COL6A3 PE=1 SV=5 | 0.868 | 0.81 |
| Q8N7G1 | Purine nucleoside phosphorylase OS=Homo sapiens PE=2 SV=1 | 0.877 | 1.324 |
| P08253 | 72 kDa type IV collagenase OS=Homo sapiens GN=MMP2 PE=1 SV=2 | 1.007 | 0.863 |
| P07333 | Macrophage colony-stimulating factor 1 receptor OS=Homo sapiens GN=CSF1R PE=1 SV=2 | 1.072 | 0.835 |
| Q9HBR0 | Putative sodium-coupled neutral amino acid transporter 10 OS=Homo sapiens GN=SLC38A10 PE=1 SV=2 | 0.554 | 0.601 |
| P11279 | Lysosome-associated membrane glycoprotein 1 OS=Homo sapiens GN=LAMP1 PE=1 SV=3 | 1.02 | 0.99 |
| H9ZYJ2 | Thioredoxin OS=Homo sapiens GN=TXN PE=2 SV=1 | 0.663 | 1.207 |
| A0A024R0V4 | Vasodilator-stimulated phosphoprotein isoform 1 OS=Homo sapiens GN=VASP PE=2 SV=1 | 0.438 | 0.616 |
| B7ZMD7 | Alpha-amylase OS=Homo sapiens GN=AMY1A PE=2 SV=1 | 0.731 | 0.932 |
| B4DR52 | Histone H2B OS=Homo sapiens PE=2 SV=1 | 1.232 | 0.867 |
| Q07954 | Prolow-density lipoprotein receptor-related protein 1 OS=Homo sapiens GN=LRP1 PE=1 SV=2 | 0.846 | 0.919 |
| A8K2W3 | cDNA FLJ78516 OS=Homo sapiens PE=2 SV=1 | 0.213 | 0.293 |
| A0A0C4DH55 | Immunoglobulin kappa variable 3D-7 OS=Homo sapiens GN=IGKV3D-7 PE=3 SV=5 | 1.047 | 0.778 |
| Q0ZCI2 | Immunglobulin heavy chain variable region (Fragment) OS=Homo sapiens PE=4 SV=1 | 1.054 | 0.847 |
| Q4ZG40 | Macrophage receptor with collagenous structure OS=Homo sapiens GN=MARCO PE=2 SV=1 | 0.995 | 1.333 |
| Q9Y2J2 | Band 4.1-like protein 3 OS=Homo sapiens GN=EPB41L3 PE=1 SV=2 | 0.585 | 1.253 |
| A8K6A6 | cDNA FLJ78619, highly similar to Homo sapiens melanoma cell adhesion molecule (MCAM), mRNA OS=Homo sapiens PE=2 SV=1 | 1.008 | 1.007 |
| P01591 | Immunoglobulin J chain OS=Homo sapiens GN=JCHAIN PE=1 SV=4 | 0.942 | 0.813 |
| Q9HDC9 | Adipocyte plasma membrane-associated protein OS=Homo sapiens GN=APMAP PE=1 SV=2 | 0.852 | 1.412 |
| P08195-4 | Isoform 4 of 4F2 cell-surface antigen heavy chain OS=Homo sapiens GN=SLC3A2 | 0.936 | 0.947 |
| Q96P63-2 | Isoform 2 of Serpin B12 OS=Homo sapiens GN=SERPINB12 | 0.739 | 1.136 |
| Q6YHK3 | CD109 antigen OS=Homo sapiens GN=CD109 PE=1 SV=2 | 0.781 | 1.111 |
| Q8TE73 | Dynein heavy chain 5, axonemal OS=Homo sapiens GN=DNAH5 PE=1 SV=3 | 0.404 | 0.959 |
| O15466-2 | Isoform 2 of Alpha-2,8-sialyltransferase 8E OS=Homo sapiens GN=ST8SIA5 | 1.019 | 1.253 |
| Q5T619 | Zinc finger protein 648 OS=Homo sapiens GN=ZNF648 PE=1 SV=1 | 1.048 | 5.144 |
| P04279 | Semenogelin-1 OS=Homo sapiens GN=SEMG1 PE=1 SV=2 | 1.153 | 1.018 |
| O00187 | Mannan-binding lectin serine protease 2 OS=Homo sapiens GN=MASP2 PE=1 SV=4 | 1.354 | 1.244 |
| B4DY90 | Tubulin beta chain OS=Homo sapiens PE=2 SV=1 | 0.589 | 0.632 |
| A0N5T0 | V-gamma-1 protein (Fragment) OS=Homo sapiens GN=V-gamma-1 PE=4 SV=1 | 1.226 | 0.594 |
| Q59GX2 | Solute carrier family 2 (Facilitated glucose transporter), member 1 variant (Fragment) OS=Homo sapiens PE=2 SV=1 | 0.971 | 1.724 |
| Q14766-4 | Isoform 4 of Latent-transforming growth factor beta-binding protein 1 OS=Homo sapiens GN=LTBP1 | 1.2 | 1.108 |
| P01344-3 | Isoform 3 of Insulin-like growth factor II OS=Homo sapiens GN=IGF2 | 0.546 | 0.697 |
| O95327 | Uncharacterized protein OS=Homo sapiens PE=2 SV=1 | 1.766 | 1.017 |
| Q9NY97 | N-acetyllactosaminide beta-1,3-N-acetylglucosaminyltransferase 2 OS=Homo sapiens GN=B3GNT2 PE=1 SV=2 | 1.244 | 1.397 |
| Q13867 | Bleomycin hydrolase OS=Homo sapiens GN=BLMH PE=1 SV=1 | 0.897 | 1.023 |
| P13473-3 | Isoform LAMP-2C of Lysosome-associated membrane glycoprotein 2 OS=Homo sapiens GN=LAMP2 | 0.987 | 0.876 |
| A1L4H1 | Soluble scavenger receptor cysteine-rich domain-containing protein SSC5D OS=Homo sapiens GN=SSC5D PE=1 SV=3 | 0.786 | 0.996 |
| A0A024R5Z9 | Pyruvate kinase OS=Homo sapiens GN=PKM2 PE=3 SV=1 | 0.719 | 0.838 |
| B2RBF5 | cDNA, FLJ95483, highly similar to Homo sapiens chitobiase, di-N-acetyl- (CTBS), mRNA OS=Homo sapiens PE=2 SV=1 | 1.109 | 1.163 |
| F6QDS0 | HCG2043426, isoform CRA_b OS=Homo sapiens GN=hCG_2043426 PE=1 SV=1 | 1.207 | 1.202 |
| O75648 | Mitochondrial tRNA-specific 2-thiouridylase 1 OS=Homo sapiens GN=TRMU PE=1 SV=2 | 0.56 | 1.237 |
| Q59FP5 | Spectrin, beta, erythrocytic (Includes spherocytosis, clinical type I) variant (Fragment) OS=Homo sapiens PE=2 SV=1 | 0.85 | 1.159 |
| Q9H9F9 | Actin-related protein 5 OS=Homo sapiens GN=ACTR5 PE=1 SV=2 | 4.634 | 1.469 |
| Q59EB6 | Complement component 1, q subcomponent, receptor 1 variant (Fragment) OS=Homo sapiens PE=2 SV=1 | 0.732 | 0.719 |
| A0A0G2JH66 | HLA class I histocompatibility antigen, alpha chain F OS=Homo sapiens GN=HLA-F PE=1 SV=1 | 1.036 | 0.985 |
| P23284 | Peptidyl-prolyl cis-trans isomerase B OS=Homo sapiens GN=PPIB PE=1 SV=2 | 0.554 | 0.803 |
| Q96MA6 | Adenylate kinase 8 OS=Homo sapiens GN=AK8 PE=1 SV=1 | 0.626 | 1.018 |
| Q4G0P3 | Hydrocephalus-inducing protein homolog OS=Homo sapiens GN=HYDIN PE=1 SV=3 | 0.796 | 1.116 |
| L7UUZ7 | Integrin beta OS=Homo sapiens GN=ITGB3 PE=2 SV=1 | 0.698 | 1.133 |
| A8K8T3 | cDNA FLJ76493, highly similar to Homo sapiens N-deacetylase/N-sulfotransferase (heparanglucosaminyl) 1 (NDST1), mRNA OS=Homo sapiens PE=2 SV=1 | 0.734 | 1.9 |
| P54725 | UV excision repair protein RAD23 homolog A OS=Homo sapiens GN=RAD23A PE=1 SV=1 | 2.66 | 2.03 |
| P55196-5 | Isoform 5 of Afadin OS=Homo sapiens GN=AFDN | 1.947 | 0.529 |
| Q65ZC9 | Single-chain Fv (Fragment) OS=Homo sapiens GN=scFv PE=2 SV=1 | 1.505 | 0.852 |
| Q9UI42 | Carboxypeptidase A4 OS=Homo sapiens GN=CPA4 PE=1 SV=2 | 0.779 | 1.008 |
| A0A1L2BU42 | Anti-staphylococcal enterotoxin A heavy chain variable region (Fragment) OS=Homo sapiens PE=2 SV=1 | 1.983 | 1.084 |
| Q8NF91 | Nesprin-1 OS=Homo sapiens GN=SYNE1 PE=1 SV=4 | 1.16 | 0.84 |
| Q15485 | Ficolin-2 OS=Homo sapiens GN=FCN2 PE=1 SV=2 | 1.02 | 0.636 |
| P47929 | Galectin-7 OS=Homo sapiens GN=LGALS7 PE=1 SV=2 | 0.88 | 1.392 |

**Supplementary Table 2:** List of plasma proteins (+1.2 -fold) upregulated during high altitude exposure (HA-D7 and HA-D150 with respect to sea level) by TMT-labeled LC-MS/MS analysis.UniPort accession number, protein description number and fold-change for each group has been mentioned.

| Accession | Description | HAD7/SL | HAD150/SL |
| --- | --- | --- | --- |
| F6QDS0 | HCG2043426, isoform CRA_b OS=Homo sapiens GN=hCG_2043426 PE=1 SV=1 | 1.207 | 1.202 |
| P19652 | Alpha-1-acid glycoprotein 2 OS=Homo sapiens GN=ORM2 PE=1 SV=2 | 1.215 | 1.228 |
| P02774-3 | Isoform 3 of Vitamin D-binding protein OS=Homo sapiens GN=GC | 3.373 | 1.237 |
| O00187 | Mannan-binding lectin serine protease 2 OS=Homo sapiens GN=MASP2 PE=1 SV=4 | 1.354 | 1.244 |
| Q08554 | Desmocollin-1 OS=Homo sapiens GN=DSC1 PE=1 SV=2 | 1.248 | 1.265 |
| P11226 | Mannose-binding protein C OS=Homo sapiens GN=MBL2 PE=1 SV=2 | 1.243 | 1.345 |
| Q7Z7Q0 | APOB protein OS=Homo sapiens GN=APOB PE=2 SV=1 | 1.327 | 1.346 |
| P02649 | Apolipoprotein E OS=Homo sapiens GN=APOE PE=1 SV=1 | 1.413 | 1.351 |
| A0A024R462 | Fibronectin 1, isoform CRA_n OS=Homo sapiens GN=FN1 PE=4 SV=1 | 1.279 | 1.365 |
| A0A024R1G8 | Apolipoprotein L, 1, isoform CRA_b OS=Homo sapiens GN=APOL1 PE=4 SV=1 | 1.423 | 1.367 |
| A0A1B1CYC5 | Vitamin D binding protein (Fragment) OS=Homo sapiens GN=Gc PE=4 SV=1 | 1.977 | 1.371 |
| Q9NY97 | N-acetyllactosaminide beta-1,3-N-acetylglucosaminyltransferase 2 OS=Homo sapiens GN=B3GNT2 PE=1 SV=2 | 1.244 | 1.397 |
| A0A024R0T8 | Apolipoprotein C-I, isoform CRA_a OS=Homo sapiens GN=APOC1 PE=4 SV=1 | 1.362 | 1.399 |
| A0A0S2Z428 | HCG2039812, isoform CRA_b (Fragment) OS=Homo sapiens GN=KRT6A PE=2 SV=1 | 1.5 | 1.408 |
| P02671 | Fibrinogen alpha chain OS=Homo sapiens GN=FGA PE=1 SV=2 | 1.773 | 1.416 |
| H6VRG1 | Keratin 1 OS=Homo sapiens GN=KRT1 PE=3 SV=1 | 1.918 | 1.452 |
| Q9H9F9 | Actin-related protein 5 OS=Homo sapiens GN=ACTR5 PE=1 SV=2 | 4.634 | 1.469 |
| Q6ZVX7 | F-box only protein 50 OS=Homo sapiens GN=NCCRP1 PE=1 SV=1 | 1.274 | 1.604 |
| P13645 | Keratin, type I cytoskeletal 10 OS=Homo sapiens GN=KRT10 PE=1 SV=6 | 1.361 | 1.61 |
| B7Z1F8 | cDNA FLJ53025, highly similar to Complement C4-B OS=Homo sapiens PE=2 SV=1 | 1.246 | 1.616 |
| B2R4P2 | cDNA, FLJ92164, highly similar to Homo sapiens peroxiredoxin 1 (PRDX1), mRNA OS=Homo sapiens PE=2 SV=1 | 1.239 | 1.655 |
| B0YIW2 | Apolipoprotein C-III OS=Homo sapiens GN=APOC3 PE=1 SV=1 | 1.976 | 1.791 |
| P54725 | UV excision repair protein RAD23 homolog A OS=Homo sapiens GN=RAD23A PE=1 SV=1 | 2.66 | 2.03 |
| Q5D862 | Filaggrin-2 OS=Homo sapiens GN=FLG2 PE=1 SV=1 | 1.994 | 2.153 |
| P00915 | Carbonic anhydrase 1 OS=Homo sapiens GN=CA1 PE=1 SV=2 | 1.253 | 2.301 |
| Q14624-2 | Isoform 2 of Inter-alpha-trypsin inhibitor heavy chain H4 OS=Homo sapiens GN=ITIH4 | 1.78 | 2.332 |
| P00918 | Carbonic anhydrase 2 OS=Homo sapiens GN=CA2 PE=1 SV=2 | 1.304 | 2.366 |

**Supplementary Table 3:** List of downregulated plasma proteins (0.8-fold) during high altitude exposure (HA-D7 and HA-D150 with respect to sea level) by TMT-labled LC-MS/MS analysis.UniPort accession number, protein description number and fold-change for each group has been mentioned.

| Accession | Description | HA-D7/SL | HAD-150/SL |
| --- | --- | --- | --- |
| A8K2W3 | cDNA FLJ78516 OS=Homo sapiens PE=2 SV=1 | 0.213 | 0.293 |
| P07737 | Profilin-1 OS=Homo sapiens GN=PFN1 PE=1 SV=2 | 0.351 | 0.349 |
| A0A024R0V4 | Vasodilator-stimulated phosphoprotein isoform 1 OS=Homo sapiens GN=VASP PE=2 SV=1 | 0.438 | 0.616 |
| P21333 | Filamin-A OS=Homo sapiens GN=FLNA PE=1 SV=4 | 0.454 | 0.558 |
| A8K3K1 | cDNA FLJ78096, highly similar to Homo sapiens actin, alpha, cardiac muscle (ACTC), mRNA OS=Homo sapiens PE=2 SV=1 | 0.468 | 0.418 |
| P63261 | Actin, cytoplasmic 2 OS=Homo sapiens GN=ACTG1 PE=1 SV=1 | 0.472 | 0.44 |
| A8K061 | cDNA FLJ77880, highly similar to Homo sapiens angiopoietin-like 3, mRNA OS=Homo sapiens PE=2 SV=1 | 0.491 | 0.514 |
| A0A1S5UZ07 | Talin-1 OS=Homo sapiens GN=TLN1 PE=2 SV=1 | 0.495 | 0.477 |
| B5BU24 | 14-3-3 protein beta/alpha OS=Homo sapiens GN=YWHAB PE=2 SV=1 | 0.502 | 0.689 |
| B7Z1K5 | Tubulin alpha chain OS=Homo sapiens PE=2 SV=1 | 0.51 | 0.463 |
| P67936-2 | Isoform 2 of Tropomyosin alpha-4 chain OS=Homo sapiens GN=TPM4 | 0.524 | 0.546 |
| P01344-3 | Isoform 3 of Insulin-like growth factor II OS=Homo sapiens GN=IGF2 | 0.546 | 0.697 |
| Q9HBR0 | Putative sodium-coupled neutral amino acid transporter 10 OS=Homo sapiens GN=SLC38A10 PE=1 SV=2 | 0.554 | 0.601 |
| P35579 | Myosin-9 OS=Homo sapiens GN=MYH9 PE=1 SV=4 | 0.562 | 0.521 |
| B4DY90 | Tubulin beta chain OS=Homo sapiens PE=2 SV=1 | 0.589 | 0.632 |
| A0A0S2Z4I5 | Complement factor properdin isoform 1 (Fragment) OS=Homo sapiens GN=CFP PE=2 SV=1 | 0.593 | 0.605 |
| V9HWK2 | Epididymis luminal protein 114 OS=Homo sapiens GN=HEL114 PE=2 SV=1 | 0.6 | 0.608 |
| P08514 | Integrin alpha-IIb OS=Homo sapiens GN=ITGA2B PE=1 SV=3 | 0.603 | 0.532 |
| E7EX29 | 14-3-3 protein zeta/delta (Fragment) OS=Homo sapiens GN=YWHAZ PE=1 SV=1 | 0.607 | 0.717 |
| P12814-4 | Isoform 4 of Alpha-actinin-1 OS=Homo sapiens GN=ACTN1 | 0.652 | 0.713 |
| A8K5T0 | cDNA FLJ75416, highly similar to Homo sapiens complement factor H (CFH), mRNA OS=Homo sapiens PE=2 SV=1 | 0.66 | 0.598 |
| P00338-3 | Isoform 3 of L-lactate dehydrogenase A chain OS=Homo sapiens GN=LDHA | 0.684 | 0.617 |
| P12273 | Prolactin-inducible protein OS=Homo sapiens GN=PIP PE=1 SV=1 | 0.687 | 0.764 |
| P03951 | Coagulation factor XI OS=Homo sapiens GN=F11 PE=1 SV=1 | 0.69 | 0.795 |
| B4E0X1 | Beta-2-microglobulin OS=Homo sapiens PE=2 SV=1 | 0.693 | 0.781 |
| P02749 | Beta-2-glycoprotein 1 OS=Homo sapiens GN=APOH PE=1 SV=3 | 0.701 | 0.756 |
| P02760 | Protein AMBP OS=Homo sapiens GN=AMBP PE=1 SV=1 | 0.708 | 0.706 |
| A0A087WVC6 | Tyrosine-protein phosphatase OS=Homo sapiens GN=PTPRJ PE=1 SV=1 | 0.713 | 0.75 |
| Q59EB6 | Complement component 1, q subcomponent, receptor 1 variant (Fragment) OS=Homo sapiens PE=2 SV=1 | 0.732 | 0.719 |
| E7EMB3 | Calmodulin-2 OS=Homo sapiens GN=CALM2 PE=1 SV=1 | 0.741 | 0.636 |
| E9KL23 | Epididymis secretory sperm binding protein Li 44a OS=Homo sapiens GN=SERPINA1 PE=2 SV=1 | 0.797 | 0.718 |
| P02747 | Complement C1q subcomponent subunit C OS=Homo sapiens GN=C1QC PE=1 SV=3 | 0.797 | 0.761 |

**Supplementary figure 1:** List of top-25 pathways identified for upregulated proteins (62 proteins, 1.2-fold) for HA-D7 group.

**Supplementary figure 2:** List of top-25 pathways identified for upregulated proteins (79 proteins, > 1.2-fold) for HA-D150 group.

**Supplementary figure 3**: List of top-25 pathways identified for downregulated proteins (< 0.08-fold, 75 proteins) for HA-D7 group.

**Supplementary figure 4**: List of top-25 pathways identified for downregulated proteins (< 0.08-fold, 56 proteins) for HA-D50 group.
